# Generation of Covalently Closed Circular DNA of Hepatitis B Viruses via Intracellular Recycling Is Regulated in a Virus Specific Manner

**DOI:** 10.1371/journal.ppat.1001082

**Published:** 2010-09-02

**Authors:** Josef Köck, Christine Rösler, Jing-Jing Zhang, Hubert E. Blum, Michael Nassal, Christian Thoma

**Affiliations:** Department of Medicine II, University Hospital of Freiburg, Freiburg, Germany; University of Southern California, United States of America

## Abstract

Persistence of hepatitis B virus (HBV) infection requires covalently closed circular (ccc)DNA formation and amplification, which can occur via intracellular recycling of the viral polymerase-linked relaxed circular (rc) DNA genomes present in virions. Here we reveal a fundamental difference between HBV and the related duck hepatitis B virus (DHBV) in the recycling mechanism. Direct comparison of HBV and DHBV cccDNA amplification in cross-species transfection experiments showed that, in the same human cell background, DHBV but not HBV rcDNA converts efficiently into cccDNA. By characterizing the distinct forms of HBV and DHBV rcDNA accumulating in the cells we find that nuclear import, complete versus partial release from the capsid and complete versus partial removal of the covalently bound polymerase contribute to limiting HBV cccDNA formation; particularly, we identify genome region-selectively opened nuclear capsids as a putative novel HBV uncoating intermediate. However, the presence in the nucleus of around 40% of completely uncoated rcDNA that lacks most if not all of the covalently bound protein strongly suggests a major block further downstream that operates in the HBV but not DHBV recycling pathway. In summary, our results uncover an unexpected contribution of the virus to cccDNA formation that might help to better understand the persistence of HBV infection. Moreover, efficient DHBV cccDNA formation in human hepatoma cells should greatly facilitate experimental identification, and possibly inhibition, of the human cell factors involved in the process.

## Author Summary

Persistent infection with hepatitis B virus (HBV) causes chronic hepatitis B which frequently progresses to hepatocellular carcinoma, a leading cause of cancer-mediated mortality worldwide. Persistence requires formation and amplification of covalently closed circular (ccc)DNA, an episomal form of the viral genome that is not targeted by current drugs and thus is responsible for the notorious difficulties in therapeutic elimination of infection. Initial generation of cccDNA occurs upon nuclear import of the virion-borne relaxed circular (rc) DNA to which the viral polymerase is covalently linked; amplification occurs via intracellular recycling. The underlying molecular pathway is poorly understood. Because HBV infects only primates, in vivo studies are extremely restricted; in vitro, select hepatoma cell lines transfected with HBV support viral replication, however with little if any cccDNA formation. Here, we compared intracellular recycling of HBV and DHBV, a model hepatitis B virus from ducks, in cross-species transfections. Surprisingly, the major contribution to cccDNA formation comes from the virus rather than the cell as DHBV but not HBV rcDNA converted efficiently into cccDNA in the same human cell background. This unexpected difference might help to better understand persistence of HBV infection; efficient DHBV cccDNA formation in human cells provides a new tool to facilitate identification, and possibly targeting, of the human cell factors involved.

## Introduction

Currently, more than 350 million people suffer from chronic HBV infection. Chronic hepatitis B frequently progresses to liver cirrhosis and hepatocellular carcinoma, a leading cause of cancer-related morbidity and mortality worldwide [1], [2].

HBV is a small enveloped hepatotropic DNA virus which replicates by reverse transcription of an RNA intermediate, the pregenomic (pg) RNA (for review: [3], [4]), to yield encapsidated, partially double-stranded rcDNA to which the viral polymerase is covalently bound [5]. Upon infection, rcDNA is transported to the host cell nucleus where it is converted into cccDNA (Figure S1). Episomal cccDNA then acts as template for all viral transcripts. These include the subgenomic RNAs encoding the surface proteins, and the pgRNA that serves as mRNA for the polymerase protein and the capsid, or core, protein. Binding of polymerase to the RNA stem-loop structure ε initiates packaging of one pgRNA molecule per newly forming capsid and its reverse transcription. The first product is single-stranded (ss) DNA of minus polarity; due to the unique protein-priming mechanism, its 5′ end is, and remains, covalently linked to the polymerase. The pgRNA is concomitantly degraded, except for its 5′ terminal ∼15–18 nucleotides which serve as primer for plus-strand DNA synthesis, resulting in rcDNA and, as a side-product, some double-stranded linear (dl) DNA. DNA-containing capsids are then enveloped by surface proteins and cellular lipids and secreted as virions. Alternatively, they are redirected to the nucleus to increase cccDNA copy number by a mechanism termed intracellular recycling; many estimates for cccDNA copies per infected hepatocyte are in the range of 5 to 50 [6]. This amplification prevents loss during cell division of the cccDNA which can not be replicated semiconservatively [7]. Thus, cccDNA formation and recycling are central to establish and maintain persistent infection, and they limit the efficacy of antiviral nucleot(s)ides in the treatment of chronic hepatitis B, as these do not directly target cccDNA (for review: [8]).

However, despite its central importance the molecular pathway driving the conversion of HBV rcDNA to cccDNA is poorly understood. In vivo studies face numerous challenges. Liver biopsies from human patients are scarce, affected by the natural history of infection, and they sample only a small volume of the liver; recent estimates for cccDNA content in infected human liver vary from 0.01 to 1.4 [9] to 0.1 to 10 copies per hepatocyte [10], with large patient-to-patient variation. Kinetic studies during acute infection, doable exclusively in chimpanzees, showed peak values of around 10 cccDNA copies per infected cell but much lower numbers before and after [11]. In well controllable experimental settings, on the other hand, such as transfected cell lines [12]–[14] or HBV-transgenic mice [15], [16], HBV produces little if any cccDNA.

DHBV is a related animal virus that is widely used as a model to study HBV infection [17]. Like HBV, DHBV produces cccDNA in cultured duck hepatocytes and in vivo [7], [18], with reported mean values of 2.9 to 8.6 copies per hepatocyte [19] though also with temporal and cell-to-cell fluctuations (from one to >36 copies per cell). Importantly, DHBV transfected into the chicken hepatoma line LMH also generates well detectable amounts of cccDNA. Initially in primary duck hepatocytes, then using that system, Summers and colleagues had first shown that knocking out surface protein expression, and thus virion secretion, dramatically increased cccDNA copy numbers per nucleus to about 200–400 molecules [20], [21]. Recent studies in stably or transiently transfected human cell lines suggest that preventing HBV surface protein expression also stimulates cccDNA formation, but to a much lower degree; instead, a “protein-free” form of rcDNA (pf-rcDNA) accumulated [12], [13]. The term protein-free (which we will adhere to here) was operationally defined by the partitioning of this rcDNA form into the aqueous phase upon phenol extraction without prior proteinase K (PK) treatment (which artificially degrades the protein); polymerase-linked DNA partitions into the organic phase. “Protein-free” does therefore not imply the complete absence of any amino acid from the DNA. Though not finally proven, several lines of indirect evidence suggest that pf-rcDNA is a precursor to cccDNA [12], [13]; not the least, removal of the bound polymerase is a *sine qua non* for cccDNA formation. Caveats are that nicked cccDNA, generated to some extent during preparation and naturally protein-free, has the same electrophoretic mobility as pf-rcDNA. Furthermore, Southern blots from infected liver nuclei have generally shown only little rcDNA versus cccDNA [22]. However, in such samples one usually looks at an established pool of cccDNA whereas the recent cell culture studies monitored initial cccDNA formation. Potential precursors accumulating under these conditions may not be detectable anymore in in vivo samples.

At any rate, the initial presence of protein-bound rcDNA inside virions and eventually of nuclear cccDNA, associated with histones [23], requires as intermediate steps nuclear transport of the rcDNA genome, its release from the viral capsid and removal of the bound polymerase to allow generation of precisely one genome length equivalents of the plus- and the minus-strand before final ligation into cccDNA (Figure S1B). The order of events is not firmly established. Intact nucleocapsids may deliver the protein-bound rcDNA to the nucleus where its release from the capsid and polymerase removal are mediated by host factors; this view is supported by the minimalistic genomes of hepadnaviruses (only ∼3 kb) and by data from nuclear transport model systems [24], [25]. Alternatively, the nucleocapsid itself may contain corresponding activities such that polymerase removal could precede capsid release, possibly already in the cytoplasm. Some evidence in favor of this view has recently been forwarded [13], [26].

Apart from these mechanistic aspects it appears, in essence, that DHBV in the avian LMH cells produces much more cccDNA than HBV in human hepatoma cells. One conceivable explanation are cell-specific differences. For instance, the routinely used human HepG2 and Huh7 hepatoma cell lines may lack enzymatic activities required for cccDNA formation that are present in the avian cells. Alternatively, the different efficiencies in cccDNA formation may be a feature of the respective viruses.

In order to address this question we took advantage of the principal ability of hepadnaviruses to replicate in hepatoma cell lines of heterologous species origin. After transfection HBV is capable of producing rcDNA in LMH cells and the same holds for DHBV in HepG2 and HuH7 cells [27], [28]. However, cccDNA formation in such cross-species transfections has not yet been addressed. Here we performed such experiments and found that, unexpectedly, the major contribution to cccDNA formation comes from the virus rather than from the cell.

## Results

### Intracellular recycling of HBV genomes occurs at very low levels

Detection of cccDNA by Southern blotting can severely be hampered by the presence of ssDNA species which have a similar electrophoretic mobility and are often present in excess. A further problem in transient transfections is the highly abundant plasmid DNA. We therefore developed an assay that essentially eliminates ssDNA and plasmid DNA by double-digestion with the restriction enzyme Dpn I and Plasmid safe DNase (PsD). Dpn I requires bacterially methylated DNA to be active and selectively restricts the transfected plasmid. PsD digests single-stranded (ss) and double-stranded linear (dl) but not circular molecules such as cccDNA. Although it has been surmised that rcDNA is a substrate for PsD [9], our own preliminary data suggested this holds only for very immature rcDNA forms. To enhance cccDNA production, we used plasmids coding for surface-deficient HBV and DHBV. Because cccDNA is enriched in the nucleus, we separated nuclei from cytoplasm by treating the cells with the mild detergent NP-40 and subsequent centrifugation. To identify encapsidated DNAs, we incubated the cytoplasmic extracts with micrococcal nuclease (MN) which digests free nucleic acids but not those protected inside capsids. Finally, because protein-bound DNA is neither recovered upon phenol extraction nor using the silica column adsorption (QIAamp) method employed here, all initial DNA preparations included a PK treatment.

**Figure 1:**
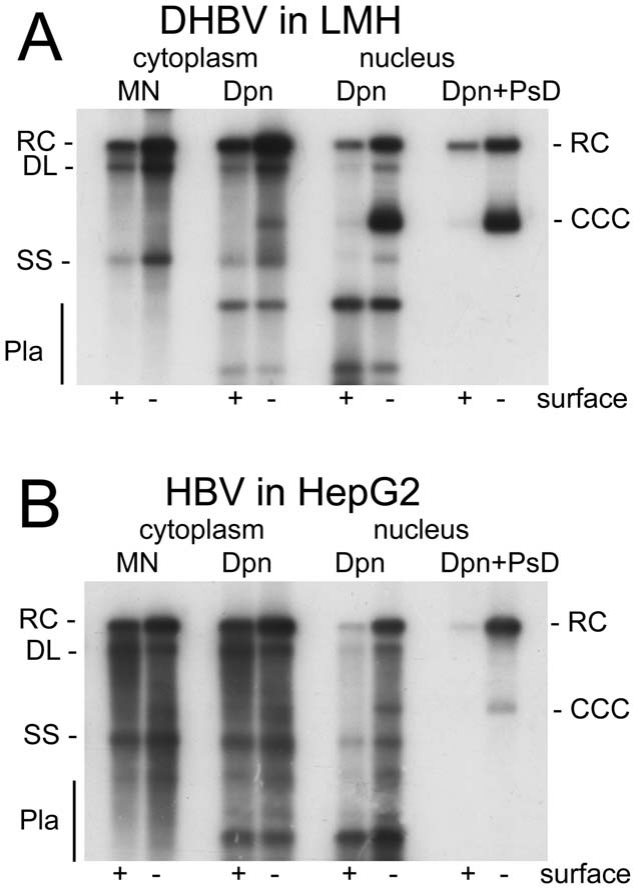
DHBV replication in avian cells and HBV replication in human cells. (**A**) Chicken LMH cells transfected with wild-type and surface-deficient DHBV. (**B**) Human HepG2 cells transfected with wild-type and surface-deficient HBV. DNAs were extracted, after prior PK digestion, from cytoplasmic lysates and the nuclear fractions. One aliquot of the cytoplasmic lysates was treated with micrococcal nuclease (MN) before DNA extraction and analyzed without further treatment. All other samples were incubated, post extraction, with either Dpn I alone (Dpn), or Dpn I plus Plasmid safe DNAse (Dpn+PsD). Nomenclature of the various DNA species: RC, relaxed circular; DL, double strand linear; SS, single strand; CCC, covalently closed circular DNA; Pla, Dpn I restriction fragments of transfected plasmid DNA.

The results for DHBV in LMH and HBV in HepG2 cells are shown in Figure 1. Treatment of the cytoplasmic samples with MN revealed the common replicative intermediates, i.e. rcDNA, dlDNA and ssDNA. Digestion with Dpn I only produced a similar pattern, except that additional plasmid-derived bands (Pla) were visible. Nuclear DNA treated with Dpn I alone produced a similar pattern, yet as expected, knock-out of surface protein expression enhanced the cccDNA signal, particularly for DHBV. Additional treatment of nuclear DNA with PsD removed all bands except those at the rcDNA and cccDNA positions; the equal signal intensities before and after PsD treatment demonstrated that mature rcDNA was not appreciably attacked by PsD. For HBV, a band at the cccDNA position was exclusively visible in the surface-deficient background (Figure 1B). However, the nuclear rcDNA signal was much more enhanced, in line with recent reports [12], [13]. Comparable results were obtained in Huh7 cells (Figure S2A). Together, these data demonstrated that the applied procedure enabled the reliable detection of cccDNA and rcDNA, without interference from other virus- or plasmid-derived nucleic acids. Furthermore, they confirmed that DHBV in avian cells produces much more cccDNA than HBV in human cells.

Faint bands with cccDNA-like mobility were also detectable in the cytoplasmic fractions of those samples containing well visible nuclear cccDNA; ethidium bromide staining of the agarose gel used to generate the blot revealed indeed some chromosomal DNA in the cytoplasmic samples, indicating an incomplete separation of two fractions. This prompted us to employ a more efficient separation procedure in later experiments (see below).

### The virus, not the cell, is the major determinant for efficient cccDNA formation

**Figure 2:**
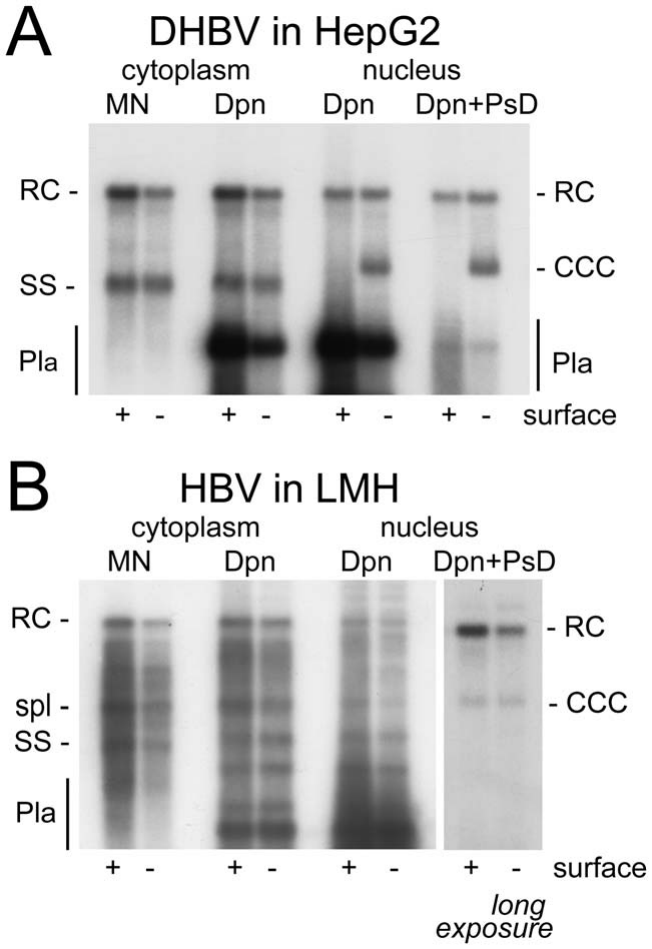
DHBV replication in human cells and HBV replication in avian cells. (**A**) DHBV in HepG2 cells. (B) HBV in LMH cells. Labeling and abbreviations are as in the legend to Figure 1. The position of a major DNA product of about 2 kb in length derived from enhanced HBV splicing in LMH cells is indicated (spl).

Next we performed analogous experiments with DHBV transfected into the human cell lines, and HBV transfected into LMH cells (Figure 2). For DHBV, cytoplasmic extracts treated with MN produced a similar pattern of replicative intermediates in HepG2 (Figure 2A) and HuH7 cells (Figure S2B) as in LMH cells (Figure 1A). HBV in LMH cells, compared to the human cell lines, generated a more complex pattern with a distinct band of an intermediate mobility between rcDNA and ssDNA (Figure 2B). This additional band originates from strongly enhanced splicing of the HBV pgRNA in the chicken cell line (Köck, Nassal, Thoma; unpublished data). The spliced genomes are of linear conformation and therefore accessible to PsD digestion. Most importantly for the current study, the envelope-deficient DHBV produced a strong cccDNA signal in both human cell lines (Figure 2A and Figure S2B), with an intensity equaling that of the nuclear rcDNA as in LMH cells. Conversely, HBV in LMH cells generated a similar pattern as in the human cell lines, with a relatively strong band at the rcDNA yet only a weak band at the cccDNA position (Figure 2B). The HBV specific lack of effect of the envelope knock-out in LMH cells correlated with a much lower abundance of the surface protein-coding mRNAs compared to the human cells (data not shown). Thus wild-type HBV in LMH cells is phenotypically similar to its envelope-deficient counterpart.

Together, these data demonstrated that both HepG2 and HuH7 cells are competent to support cccDNA synthesis, yet with strikingly higher efficiency for DHBV than HBV; conversely, LMH cells did not support more efficient cccDNA formation for HBV. These results were reproduced in about twenty independent experiments. Furthermore, this virus-specific difference was neither changed by increasing pgRNA levels via replacement of the HBV core promoter by the CMV promoter, nor by analyzing the cultures at earlier or later time points post transfection (Figure S3). Thus the virus contributes more profoundly to the efficiency of cccDNA biosynthesis than the cell. More quantitative data on the amounts and intracellular distribution of the different viral DNA were obtained with samples from gradient purified nuclei.

### Purification of cell nuclei by sucrose gradient sedimentation

Beyond the markedly different levels of cccDNA, the data shown above also revealed distinct levels of nuclear rcDNA, and of the ratios of cccDNA to rcDNA, between DHBV and HBV. A detailed characterization of the nuclear rcDNA was expected to provide clues on the rate-limiting step of HBV cccDNA synthesis. This, however, required the nuclear preparations to be as free from cytoplasmic contamination as possible. We therefore separated the nuclei from the cytoplasm by sucrose density sedimentation [29]. Western blot analyses of nuclear extracts versus total cell lysates showed that, while nuclear histone H3 was detected in either fraction, the cytoplasmic poly(A) binding protein (PABP) was exclusively present in total cell lysates but not in nuclear extracts (Figure S4A). Co-purification of cytoplasmic capsids with the nuclei was ruled out by the absence of viral DNAs from purified nuclei of non-transfected HepG2 cells that had been mixed with capsid-containing cytoplasmic fractions from HBV- or DHBV-transfected cells and subjected to the same procedure (Figure S4B). Permeability of the nuclei for exogenously added MN, an assay subsequently used to address nuclease sensitivity vs. resistance of the nuclear viral DNAs, was confirmed by dose-dependent generation of chromosomal DNA fragments with sizes of multiples of ∼150 bp, as expected from cleavage between nucleosomes (Figure S4C).

### Less efficient nuclear transport of HBV versus DHBV rcDNA may contribute to but does not solely explain less efficient cccDNA accumulation

**Figure 3:**
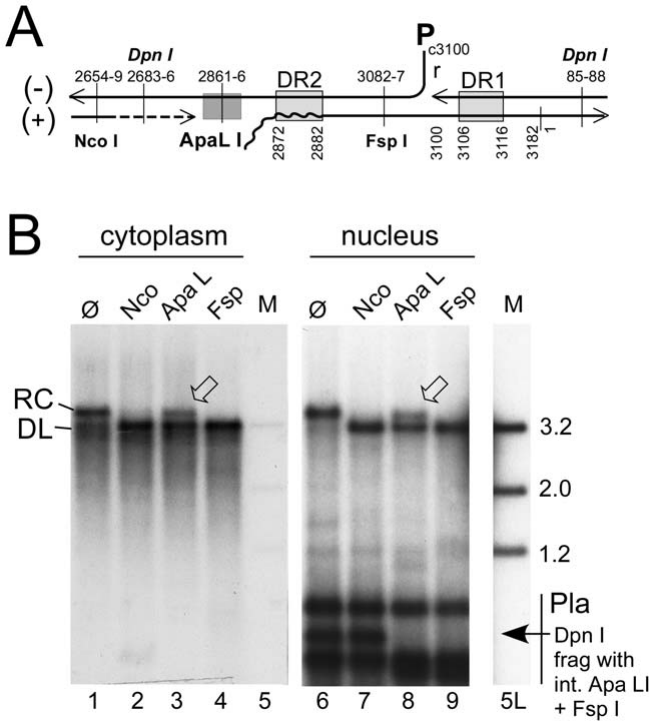
Site-specific discontinuity confirms the rcDNA nature of a substantial fraction of nuclear HBV DNA. Extensive nicking of HBV cccDNA might pretend an artifactually high ratio of rcDNA to cccDNA in the nucleus. However, nicking should occur at random whereas RC-DNA is distinctly discontinuous where the minus-strand and plus-strand DNA start. (**A**) Scheme of HBV rcDNA discontinuities. Restriction site positions are indicated with the first and last nucleotide of the recognition sequences. The Apa LI site is located immediately upstream of the plus-strand start. DNA in which the plus-strand is not sufficiently extended can not be cut. DR1 and DR2, direct repeats 1 and 2; wavy line at DR2, RNA primer at plus-strand 5′ end. (**B**) About one third of the rcDNA signal is resistant to Apa LI but not Nco I or Fsp I digestion. Cytoplasmic (treated with MN) and nuclear (treated with Dpn I) DNA preparations (both after prior PK digestion) were incubated with the indicated restriction enzymes. Consistently (Figure S5B), ∼35% of the rcDNA signal from the cytoplasm as well as the nucleus remained upon incubation with Apa LI (arrowheads) but not Nco I or Fsp I. Activity of Apa LI in the reactions is documented by the absence of a plasmid-derived Dpn I fragment containing internal sites for Apa LI and Fsp I but not Nco I. All samples were run on the same gel but a six-times longer exposure is shown for the nuclear samples; lane 5L on the longer exposure corresponds to lane 5 on the left panel. M, marker fragments of the indicated sizes (in kb).

Applying this methodology to HepG2 cells transfected with surface-deficient HBV or DHBV constructs revealed that the purified nuclei contained easily detectable signals at the rcDNA position, and for DHBV a strong and for HBV a weak signal at the cccDNA position; as expected, both converged into a single species with dlDNA mobility upon digestion with Eco RI, and heating converted the rcDNA but not the cccDNA signal into a new band with ssDNA mobility (Figure S5A). However, this assay does not discriminate true rcDNA from randomly nicked cccDNA. For distinction, we exploited the defined discontinuities at the 5′ ends of the complete minus-strand and the 3′ terminally incomplete plus-strand DNA (Figure 3A) in rcDNA and the requirement of most restriction enzymes for a double-stranded substrate structure. The nuclear, and for comparison also the cytoplasmic, HBV DNA preparations were incubated with Nco I whose recognition site (CCATGG; nt positions 2654–2659) is in the 3′ proximal part of the plus-strand but largely double-stranded already in virion DNA (e.g. [30]); Fsp I (TGCGCA; nt positions 3082–3087), recognizing a site in the 5′ proximal plus-strand region that is double-stranded even in DNA with very short plus-strands; and Apa LI (nt positions 2861–2866), with its recognition site (GTGCAC) ending only 5 nt upstream of DR2 (nt positions 2872–2882) where the plus-strand begins. Nicked cccDNA should be linearized by all three enzymes whereas true rcDNA, depending on how far the plus-strand is filled-in, is expected to be partially or completely resistant to Apa LI cleavage. Exactly this was observed, with about 35% of the rcDNA remaining in the Apa LI but not the Nco I and Fsp I treated samples from both the cytoplasm and the nucleus; very similar results were obtained in repeat experiments (42.2±10.7% for the nuclear and 40.0±2% for the cytoplasmic rcDNA), as well as for protein-free nuclear rcDNA (32.0±4.9%; Figure S5B). The differences between different treatments were highly significant (P<0.05 to <0.001) but those between cytoplasmic vs. nuclear rcDNA were not. Activity of Apa LI in the reactions was demonstrated by the disappearance of the Dpn I fragment from the transfected plasmid DNA that harbors the single virus genome-encoded recognition sites for Apa LI and Fsp I but not Nco I. Furthermore, an admixed DHBV plasmid was completely cut by either enzyme (Figure S5B). Hence, in accord with previous reports [12], [13], a substantial fraction of the nuclear HBV rcDNA signal was derived from true rcDNA, confirming that the weak cccDNA signal was not caused by excessive nicking.

**Figure 4:**
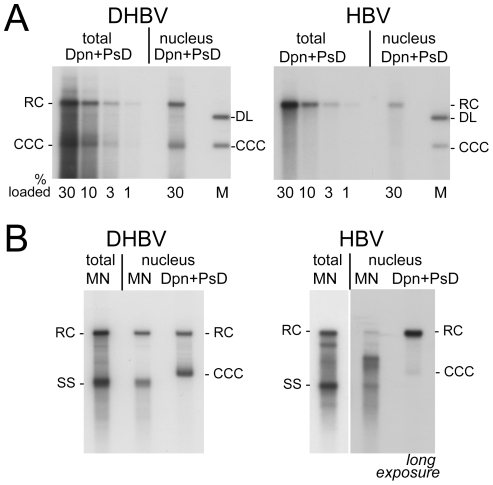
Intracellular distribution and nuclease sensitivity of viral DNAs. HepG2 cells were transfected with vectors for surface-deficient DHBV (left panels) or HBV (right panels). (**A**) Relative nuclear distribution. DNA was extracted, after prior PK treatment, from total cells or from gradient-purified nuclei and subsequently digested with DpnI plus PsD. Serially diluted samples were loaded on the gel. Loading volumes are indicated in percent of the total sample volume, obtained from one well of a 6-well plate. One of three experiments used for quantification (see text for details) is shown. (**B**) Direct comparison of MN resistant versus total nuclear viral DNA. DNA was prepared from total cell extract treated with MN plus PK (MN), or from gradient-purified nuclei; equal aliquots of the nuclei were treated with MN plus PK before DNA extraction (MN), or with only PK followed by incubation with Dpn I plus PsD (total nuclear DNA). A six times longer exposure for the nuclear HBV samples is shown to better reveal weak signals. Quantification indicated that only ∼10% of the nuclear full-length HBV versus ≥80% of the nuclear DHBV DNA were MN resistant.

For a quantitative estimate of the proportion of nuclear versus cytoplasmic HBV and DHBV rcDNA, we compared the amounts of viral DNA in total cells (nuclei plus cytoplasm) and in gradient-purified nuclei; to account for all DNA species regardless of encapsidation and protein-linkage status, preparations involved PK treatment and subsequent digestion with Dpn I plus PsD, but not MN. Serial dilutions served to improve the accuracy of quantitation by phosphorimaging; one of three independent experiments used for quantitation is shown in Figure 4A. The signals from the four different amounts of total rcDNA (1%, 3%, 10%, and 30% of the whole preparation) and from the 30% portions of nuclear rcDNA were quantitated and corrected for background by phosphorimaging. Accordingly, the ratios for total vs. nuclear rcDNA were 3.6±0.44 : 1 for DHBV, and 6.8±0.26 : 1 for HBV, i.e. about 25–30% of the DHBV and around 15% of the HBV rcDNA were nuclear.

Using the known amounts of HBV and DHBV marker DNAs run on the same gels we also estimated the amounts per sample (one well of a 6-well plate) of total versus nuclear rcDNA and cccDNA, and the copy numbers per transfected cell (see Text S1 for details); accordingly, DHBV generated ∼160 pg (161.6±28.3; 200±35 copies) total rcDNA of which ∼45 pg (44.8±4.5; 56±6 copies) were nuclear, and HBV produced ∼130 pg (130.2±7.6; 162±10 copies) total rcDNA of which ∼20 pg (19.2±1.1; 25±2 copies) were nuclear. Amounts of cccDNA estimated analogously were ∼45 pg (42.3±3.3; 56±5 copies) for DHBV, and ∼2 pg (2.1±0.9; 2.6±1.1 copies) for HBV; cccDNA values for HBV were close to background and therefore difficult to determine more accurately. However, a similar excess of nuclear HBV rcDNA over cccDNA has also been reported for inducible cell lines stably transfected with surface-deficient HBV [12], [13]. Together these data indicated that less efficient nuclear transport of HBV rcDNA contributes to, but cannot solely be responsible for, the much less efficient cccDNA accumulation.

### Nuclear HBV but not DHBV full-length rcDNA is highly accessible to exogenously added nuclease

To further investigate the status of the viral DNAs, we treated total lysates and purified nuclei with MN followed by PK, leaving only encapsidated (or otherwise protected) DNA species intact. For DHBV, the results were essentially the same (Figure S6, panel DHBV) as those after Dpn I plus PsD treatment (Figure 4A) but for HBV the signal at the full-length rcDNA position had nearly disappeared, apparently in favor of faster migrating species (Figure S6, panel HBV). For a direct estimate of the fraction of MN sensitive nuclear DNA species, viral DNA was isolated from equally sized aliquots of the nuclei by either the Dpn I plus PsD or the MN procedure and analyzed side-by-side (Figure 4B). For DHBV the total amounts of rcDNA obtained in either way were very similar, indicating that most of the nuclear DHBV DNA was present in largely intact nucleocapsids (as confirmed by anti-capsid immunoprecipitation; see below). The nonetheless strong cccDNA signal suggested that once released from the capsid, DHBV rcDNA gets rapidly converted into cccDNA; alternatively, the stably encapsidated nuclear DHBV rcDNA might be a dead-end product.

For HBV, in contrast, only the cytoplasmic rcDNA signal was largely resistant to MN whereas in the nuclear sample faster migrating species accumulated (Figure 4B and Figure S6); quantitative comparison of the nuclear full-length rcDNA signals with versus without MN treatment from three independent transfections showed that only about 10% (11.1±6.3%) of the full-length rcDNA was resistant. Hence different from DHBV, most of the nuclear HBV rcDNA was sensitive to MN and therefore no more protected by an intact capsid shell.

### Core protein association of nuclease sensitive nuclear HBV rcDNA indicates partial disintegration of the HBV capsid in the nucleus

The nuclease sensitivity of most of the nuclear HBV full-length rcDNA was compatible with its complete release from the capsid; the faster-migrating nuclease resistant species might then represent shorter DNAs that were still fully encapsidated. Alternatively, partial opening of the capsid could have exposed parts of, but not the entire full-length DNA to nuclease attack; finally, partial protection could also have arisen from association with factors other than core protein.

We therefore assessed whether the nuclear viral DNAs could be immunoprecipitated by antibodies against the respective core proteins. Purely osmotic procedures released much less core protein from the gradient-purified nuclei than lysis with 0.5% SDS which, however, disintegrates capsids and prevents analysis of capsid-associated nucleic acids. Instead we treated the nuclei with 0.75× radioimmunoprecipitation (RIPA) buffer which destroys the nuclear envelope but leaves the viral nucleocapsids intact [31]. The cytoplasmic lysates serving as reference source for the immunoprecipitations (IPs) were likewise adjusted to 0.75× RIPA. As a specificity control, HBV samples were incubated with anti-DHBV core antibody and vice versa (mock-IP). Next, DNAs associated with the immunopellets were isolated after prior PK treatment and analyzed by Southern blotting.

**Figure 5:**
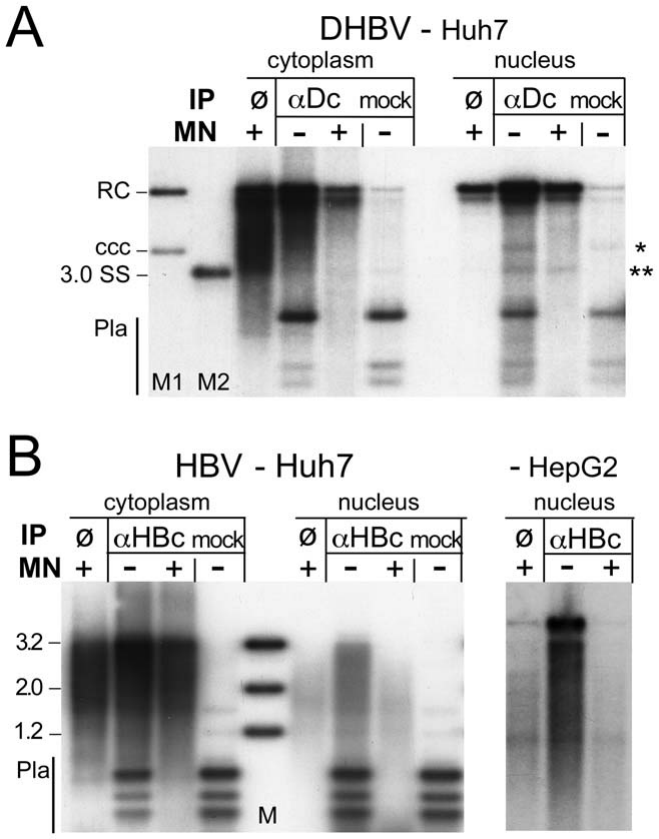
Core protein association of nuclear DHBV and HBV DNA. Vectors for surface-deficient DHBV and HBV genomes were transfected into Huh7 cells. IPs were performed in cytoplasmic extracts and extracts of purified nuclei containing 0.75× RIPA buffer, using antibodies against DHBV core protein (αDc) or HBV core protein (αHBc); in the mock IPs αDc was replaced by αHBc and vice versa. Immunopellets were treated with MN or not as indicated, and extracted after prior PK digestion. Purified DNAs from not MN-treated samples were digested with Dpn I. ø, extract directly treated with MN. (**A**) DHBV. M1, marker for cccDNA and rcDNA; M2, marker for single-stranded DNA; * and **, positions of cccDNA and ssDNA, respectively. (**B**) HBV. The rightmost panel shows the nuclear samples from an analogous experiment in HepG2 cells; the cytoplasmic samples are shown in Figure S7.

For DHBV (Figure 5A), the immunoprecipitated DNA from both the cytoplasm and the nuclei, if treated with only Dpn I, generated a similar pattern as that obtained by direct incubation of the extracts with MN (lanes ø), except it contained some fragmented plasmid DNA; this was found in similar amounts in the mock-IP and represented at most 50 pg, i.e. a tiny fraction of the transfected plasmid DNA. MN treatment of the immunopellets completely removed the residual plasmid DNA, as well as a faint band at the cccDNA position seen only in the nuclear immmunopellets, but left most of the viral rcDNA and dlDNA intact. Hence the non-ccc forms of DHBV DNA in the cytoplasm and nucleus behaved alike: both were immunoprecipitable with anti-DHBV core antibody and both were largely protected from nuclease, consistent with their being stably encapsidated in either compartment.

For HBV (Figure 5B), the picture in the cytoplasmic samples was similar; the anti-HBV core antibody precipitated the same type of DNAs as obtained by direct MN treatment; residual plasmid DNA was completely removed by MN. The nuclear immunopellet, if treated with Dpn I only, generated a similar pattern as that from the cytoplasm; hence at least part of the nuclear full-length rcDNA was still core protein-associated. However, like direct incubation of the nuclei with MN (Figure 4B), MN treatment strongly reduced the full-length rcDNA signal whereas faster migrating DNA species accumulated, suggesting their resistance was indeed due to protection by the capsid. Comparable results were obtained in Huh7 (Figure 5B, left panel) and in HepG2 cells (Figure 5B, right panel; cytoplasmic samples of this experiment are shown in Figure S7). Together these data indicated that at least a fraction of the nuclear HBV DNA including full-length species was still associated with core protein but, different from DHBV, was no more fully protected.

### Restriction of partially nuclease-resistant nuclear HBV DNA suggests region-selectively opened nucleocapsids as a putative uncoating intermediate

**Figure 6:**
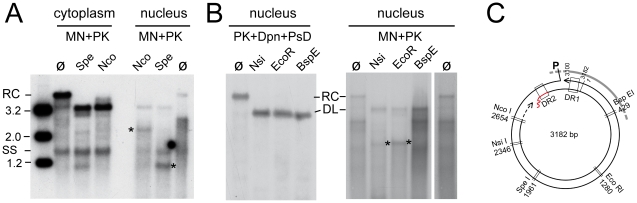
Restriction mapping of nuclear HBV rcDNA suggests genome region-selective MN accessibility. (**A**) Cytoplasmic versus nuclear MN-resistant viral DNAs. DNAs were isolated from HepG2 cells transfected with the surface-deficient HBV vector after prior MN plus PK treatment, and incubated with restriction enzymes Spe I and Nco I (ø, no restriction). Amounts equivalent to 10% of the total cytoplasmic fraction and 90% of the total nuclear fraction were loaded. (**B**) Total nuclear rcDNA versus MN resistant nuclear DNA. Viral DNA from isolated nuclei was prepared by either the PK plus Dpn I plus PsD procedure (total nuclear rcDNA), or after prior MN plus PK treatment (MN resistant nuclear DNA), and incubated with Nsi I, Eco RI or Bsp EI. Asterisks denote newly formed distinct fragments; lane ø on the right is a longer exposure of lane ø on the left. (**C**) Restriction map of HBV. The restriction sites probed in A and B are indicated. DR1 and DR2, direct repeats 1 and 2; P, covalently linked polymerase; wiggly red line, RNA primer at (+)-DNA 5′ end. Together, the patterns can consistently be explained if MN treatment removed a defined region from the rcDNA that approximately encompasses position 3000 to 500.

The majority of MN resistant nuclear HBV DNAs migrated reproducibly to a region whose upper boundary would correspond to dlDNA of ∼2.4–2.7 kb (Figure 4B). One explanation was that these molecules represented naturally shorter DNA derived from spliced pgRNA [32], [33] which remained protected in intact nucleocapsids. However, a construct encoding a variant HBV genome in which the major splice acceptor site was mutated generated exactly the same pattern of MN resistant nuclear DNA although splicing was indeed suppressed (data not shown). Next we incubated the MN resistant nuclear, and for comparison also cytoplasmic, HBV DNA with restriction enzymes Nco I and Spe I which cut the HBV genome uniquely at positions 2654 and 1961 (Figure 6A, C). Expectedly, the major effect on the cytoplasmic DNA was conversion of the rcDNA to dlDNA, plus generation of small amounts of fragments that most likely derived from dlDNA; ssDNA was not affected. In the nuclear sample, the little full-length rcDNA was linearized as well. The major faster migrating species disappeared completely upon exposure to either enzyme, indicating they contained both recognition sites in double-stranded form. Intriguingly, within the background smear distinct fragments of about 2.2 kb (Nco I) and 1.2 kb (Spe I) appeared that were absent from the non-restricted sample, suggesting that MN had generated at least one relatively distinct new DNA end at a fixed distance from the restriction sites. One interpretation was that the MN products of rcDNA lacked about 500 bp roughly between position 3000 and 500 (see map in Figure 6C); in that case the second Nco I fragment would comprise only around 300 to 400 bp which, together with some heterogeneity in size, would make it difficult to detect. The 1.2 kb band in the Spe I treated sample, conversely, could consist of two about equally sized products. To corroborate this assumption, we digested the nuclear MN resistant DNAs with three more single cutter enzymes (Figure 6B): Nsi I and Eco RI (recognition sites starting at positions 2346 and 1280, respectively), and Bsp EI (recognition site starting at position 429 in the predicted lacking sequence part). As a further control, intact nuclear rcDNA was isolated by the Dpn I plus PsD procedure and digested with the same enzymes which in all cases led to complete linearization (Figure 6B, left panel). In the MN treated nuclear DNA, the rcDNA signal likewise disappeared completely in favor of a new band at the dlDNA position. Importantly, however, Nsi I and Eco RI produced distinct new bands of about 1.9 kb (marked by *), whereas for Bsp EI the pattern below the dlDNA position was not detectably different from that of the untreated sample (lanes ø). These data are consistent with MN generating from nuclear HBV rcDNA a relatively distinct mixture of double-stranded linear DNAs that lack approximately the region between position 3000 and 500 (Figure 6C). This, in turn, implies that capsid opening occurs at distinct sites relative to the packaged genome.

Together, these data indicated that nuclear HBV genome release from the capsid is efficiently initiated; at most 10% of the full-length rcDNA remained resistant to MN. Furthermore, as shown below, we also found evidence for a fraction of nuclear rcDNA that is completely released from the core protein. Hence poor HBV rcDNA to cccDNA conversion is not caused by a complete block of uncoating dynamics.

### The majority of nuclear HBV full-length rcDNA is not linked to viral polymerase protein

**Figure 7:**
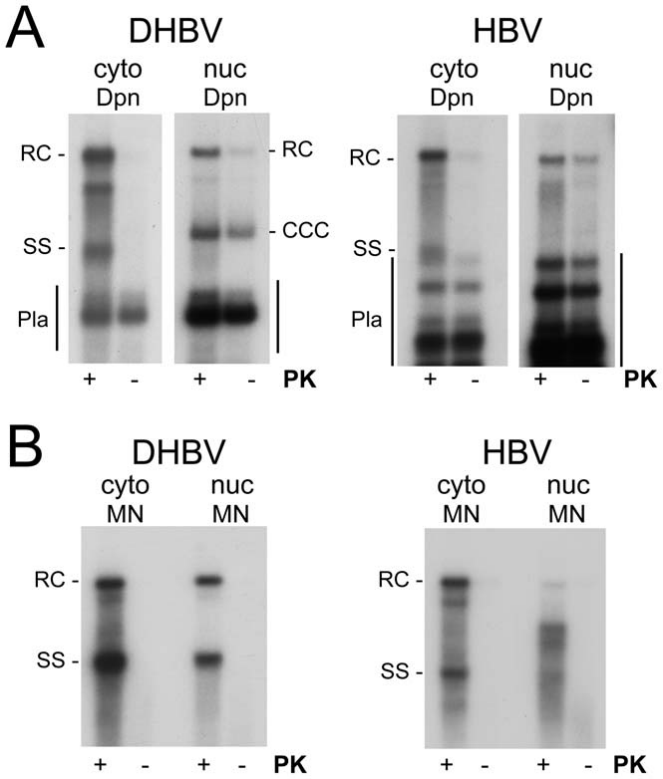
Polymerase linkage status of cytoplasmic versus nuclear viral DNAs. HepG2 cells were transfected with vectors for surface-deficient DHBV (left panels) or HBV (right panels). (**A**) Full-length rcDNA. DNA was extracted from cytoplasmic lysate (cyto) or gradient-purified cell nuclei (nuc) by phenol extraction with or without prior PK treatment (+/− PK) and subsequently incubated with Dpn I. For HBV small amounts of partial Dpn I digestion products extended up to close to the position of ssDNA (Pla). Note the comparably strong signal for nuclear, but not cytoplasmic, HBV rcDNA even without PK treatment. (**B**) Nuclease resistant DNAs. Cytoplasmic lysate and gradient-purified nuclei were treated with MN. Subsequently, DNA was prepared by phenol extraction with or without prior PK digestion. All samples not treated with PK produced only weak if at all detectable signals.

To test the polymerase linkage status of the different viral DNAs we prepared cytoplasmic extracts and purified nuclei from DHBV and HBV transfected HepG2 cells. Equal aliquots from each sample were then incubated in buffer containing SDS with or without PK, and subjected to conventional phenol extraction. Transfected plasmid DNAs were digested with Dpn I (Figure 7A). Without PK, only very weak signals were seen for both HBV and DHBV in the cytoplasmic fractions (<10% of the signals with PK), in line with a previous report [12] though not with an other [13]; evaluation of 15 independent HBV samples gave a mean value of 9.2±2.4%.

In the nuclei of the DHBV transfected cells, PK treatment had, expectedly, little effect on cccDNA and the plasmid derived Dpn I fragments (Figure 7A, left panel); but strongly enhanced the rcDNA signal. For nuclear HBV rcDNA, in contrast, the signals obtained without PK were nearly as strong as those from the PK treated aliquot (Figure 7A, right panel). For a semiquantitative estimate we compared the rcDNA signal intensities with versus without PK treatment from two independent experiments; to account for differencies in recovery, these values were normalized for the cccDNA signals (DHBV) and the plasmid fragments (HBV), respectively, in the same lanes. Accordingly, the rcDNA signals from the PK treated samples were 3.78±0.54 fold stronger for DHBV, and 1.17±0.29 fold stronger for HBV, indicating that around 20% of the nuclear DHBV rcDNA and around 70% or more of the nuclear HBV rcDNA were protein-free. Comparing 11 different pairs of PK treated vs. non-treated nuclear HBV samples yielded a mean value of 66.9±13.3% protein-free rcDNA.

That a major fraction of the rcDNA signals originated from true rcDNA was corroborated by subjecting nuclear HBV DNA obtained with or without prior PK treatment to digestion with Nco I, Apa LI, and FspI. As before (Figure 3), Nco I and Fsp I converted the PK treated rcDNA nearly completely into dlDNA, whereas about 40% of the rcDNA signal remained upon Apa LI digestion (Figure S5B); for the protein-free nuclear DNA the mean value was slightly lower (32±5%), suggesting it contained more completely filled-in plus-strand DNA. However, this difference was not statistically significant.

We also assessed the polymerase linkage status of the MN resistant DNAs (Figure 7B). The patterns generated upon prior PK treatment fully matched those previously seen (Figure 4B, C, S6) whereas without PK treatment only faint signals were detectable. For DHBV, the nuclear and cytoplasmic DHBV DNAs were similar in composition and both were largely polymerase-linked. With, but not without PK treatment, nuclear HBV DNA showed the same accumulation of faster migrating species as before (Figure 4B). Hence the MN resistant shorter HBV DNA species as well as the small amounts of resistant full-length rcDNA were mostly protein-linked. This suggested that the relatively high proportion of protein-free species in the total nuclear DNA (Figure 7A) was largely accounted for by MN sensitive rcDNA molecules.

### Evidence for a fraction of nuclear HBV full-length rcDNA that is completely released from the capsid

The IP experiments described above (Figure 5B) indicated an association of at least some of the nuclear HBV full-length rcDNA with core protein. Hence failure to completely uncoat the DNA could represent a rate-limiting step in cccDNA formation. We therefore assessed whether the nuclei also contained rcDNA molecules that were no more associated with core protein. To this end, we performed IPs as before yet this time we included the IP supernatants in the analysis. Furthermore, we addressed the polymerase linkage status of such putative species by treating one half of each sample with PK, the other not. Cytoplasmic samples in which the viral DNA is largely present in intact capsids (Figure 5) served as control. Because MN would destroy nonprotected rcDNA, we used Dpn I plus PsD to reduce the background of non-rcDNAs.

**Figure 8:**
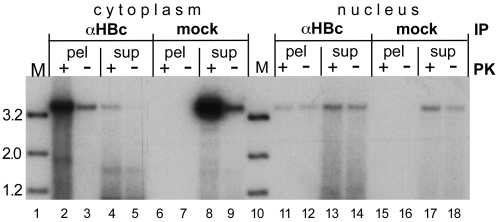
Evidence for a fraction of completely uncoated, largely protein-free nuclear HBV RC-DNA. HepG2 cells were transfected with the vector for surface-deficient HBV. IPs were performed as in Figure 5, except that DNA from both the immunopellets (pel) and the supernatants (sup) was analyzed; in addition, the samples were treated, or not treated, with PK prior to DNA extraction as indicated. Note the high proportion of rcDNA in the αHBc supernatant from the nuclear vs. cytoplasmic sample, and the likewise high proportion of rcDNA signals in the nuclear vs. cytoplasmic samples not treated with PK.

The specific IP from the cytoplasm generated a strong rcDNA signal in the immunopellet after PK treatment; the signal from the supernatant was only 3–4% as intense (Figure 8, lane 2 vs. 4). Conversely, the signal from the mock IP pellet had less than 3% the intensity of that from the supernatant (lane 6 vs. 8). These data confirmed that the IP was specific and that the amount of anti-HBc antibody was sufficient to precipitate ≥95% of the cytoplasmic rcDNA. The strongly reduced signals without PK treatment further corroborated that 90% or more of the cytoplasmic rcDNA was protein-linked.

A different picture was seen in the nuclear samples. The anti-HBc supernatant contained more rcDNA than the immunopellet (lane 11 vs. 13); quantification indicated that only about 30% of the rcDNA was precipitated (29.8±3.5% for the PK treated, 34.2±3.2% for the not treated sample; from duplicate determinations of two independent experiments). For the mock IP, only 5.0±3.9% were found in the pellet (lane 15 vs. 17). In further contrast to the cytoplasmic samples, yet in line with the previous data (Figure 7A), omitting the PK treatment only modestly reduced the signal intensities in all nuclear samples (lanes 11 vs. 12; 13 vs. 14; 17 vs. 18), i.e. to 73±16% in the specific immunopellet, to 69±9% in the supernatant, and to 55±7% in the mock IP supernatant. Although the low overall signal intensities precluded a more accurate determination, these data indicated that around two thirds of the nuclear HBV rcDNA were not stably associated with core protein, i.e. probably completely uncoated, and around two thirds of these molecules were no more linked to intact polymerase protein.

### Completion of plus-strand DNA in serum-derived HB and DHB virions is not sufficient for capsid release or polymerase protein removal from rcDNA

The data described above were compatible with at least partial release of the nuclear HBV rcDNA from the capsid, allowing cell factors to engage in polymerase removal. A recently proposed alternative is that polymerase removal might precede capsid opening, possibly already in the cytoplasm and mediated by capsid-intrinsic activities [26]. One line of evidence in favor of this proposal was the reported generation of small amounts of protein-free DNA in detergent-stripped DHB virions subjected to prolonged endogenous polymerase reaction (EPR) conditions; no results for HB virions were reported. In the EPR, exogenously added dNTPs are utilized by the capsid-borne (“endogenous”) polymerase to complete the plus-strand DNA. Because serum virions are secreted from infected hepatocytes, there is very little risk of cross-contamination with nuclear or other cellular factors.

**Figure 9:**
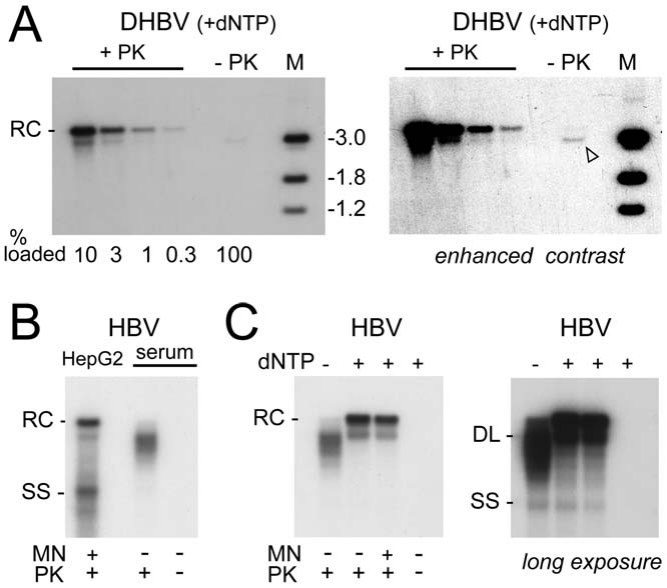
Efficient plus-strand DNA extension does not induce uncoating and polymerase removal from rcDNA. Nucleocapsids were obtained by detergent-stripping of virions from highly viremic sera and subjected to a 16 h endogenous polymerase reaction (EPR; +dNTP) as described [26]. (**A**) DHBV. After the EPR, viral DNA was prepared via phenol extraction with or without prior PK treatment. All of the non-treated and the indicated fractions of the PK-treated sample were loaded on a gel and analyzed by Southern blotting. A very long exposure plus contrast enhancement (right panel) revealed a faint band at the dlDNA but not the rcDNA position. (**B**) Comparison of electrophoretic mobility of transfection-derived and virion-derived HBV DNA. Cytoplasmic DNA from transfected HepG2 cells and virion DNA (prior to EPR) was treated as indicated, isolated via phenol extraction, and analyzed by Southern blotting. Note the faster mobility of most virion-derived DNAs, indicative of incompletely extended plus-strands, and the virtual absence of signal without prior PK treatment. (**C**) HBV EPR products. Nucleocapsids subjected to long-term EPR conditions were treated as indicated. The shift towards the positions of full-length rcDNA and dlDNA after the EPR indicated that the nucleocapsid-borne polymerase was active. Nearly all DNA was resistant towards MN. Even an overexposed autoradiogram (right panel) did not reveal a signal without prior PK treatment.

Here we performed analogous experiments with HB virions, and for comparison with the published results [26] also with DHB virions. Nucleocapsids of either virus were obtained from highly viremic sera by sedimentation in Nycodenz gradients containing NP-40 detergent. DHBV nucleocapsids were exposed to EPR conditions for 16 h [26], and the capsid-borne DNAs were isolated via phenol extraction with or without prior PK treatment. The corresponding Southern blot showed ample rcDNA and some dlDNA in the PK treated but not the untreated sample (Figure 9A, left panel), confirming that most of the virion-borne genomes are covalently linked to polymerase. In line with the reported data, a long exposure plus contrast enhancement (Figure 9A, right panel) revealed indeed a faint band of apparently protein-free DNA, however exclusively at the dlDNA, not the rcDNA position. By comparison with the dilution series of the PK treated sample, the protein-free dlDNA accounted for less than 0.3% of the total capsid-borne virus DNA.

Analysis of the HB virion-derived nucleocapsids after PK treatment but prior to the EPR (Figure 9B) showed a smear of bands that migrated faster than mature rcDNA from transfected HepG2 cells, indicating a relatively large gap in the HB virion plus-strand DNA; no signal was visible without PK treatment (Figure 9B). When subjected to EPR (Figure 9C, lanes +dNTP) under the same conditions as used for DHBV, these species were efficiently converted into rcDNA plus some dlDNA, confirming enzymatic activity of the capsid-borne polymerase. The signal was largely stable towards MN, indicating the vast majority of viral genomes remained protected by the capsid shell. Without PK treatment, no signal was visible, not even upon overexposure (Figure 9C, right panel). Thus if plus-strand completion *per se* caused any release from the capsid or removal of polymerase from viral DNA, its extent for HBV was even less pronounced than for DHBV. That, in contrast, in the nuclei of the transfected cells most of the HBV rcDNA was sensitive to MN and protein-free (Figure 4, 5) strongly supports that HBV capsid opening and deproteinization of rcDNA depend largely on the nuclear environment.

## Discussion

**Figure 10:**
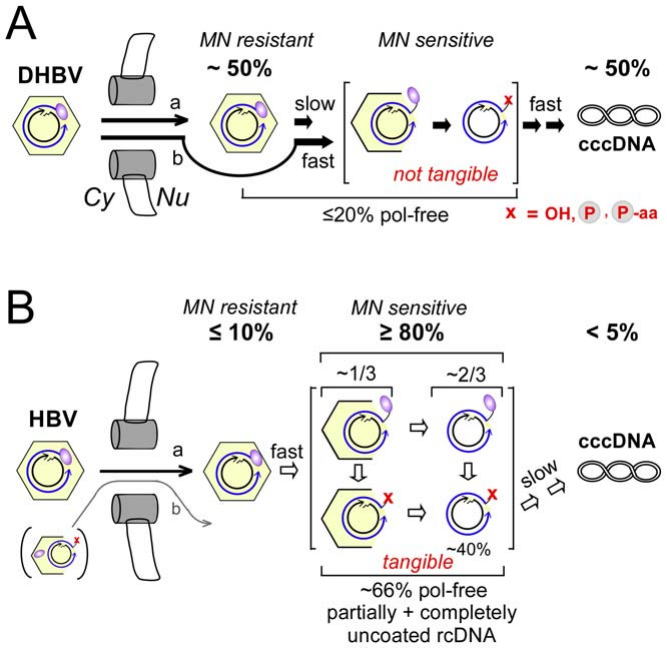
Virus-specific differences between DHBV and HBV cccDNA formation. The cartoon summarizes the steady-state levels of different rcDNA forms in human HepG2 cells transfected with surface-deficient viruses. They suggest specific steps that differentially affect the rate of cccDNA formation. Cytoplasmic rcDNA of both viruses was largely present in intact nucleocapsids, with <10% in protein-free form. The about four-fold higher nuclear import efficiency of DHBV vs. HBV nucleocapsids is not considered. The red x indicates absence of intact polymerase; the minus-strand 5′ end may consist of a hydroxyl (OH) or phosphate (encircled P) group, or still contain one or more amino acid residues (aa) from the polymerase. (**A**) DHBV. Nuclei contained ∼50% rcDNA plus ∼50% cccDNA. Most of the rcDNA was present in MN resistant, i.e. bona fide intact, capsids. The fraction of protein-free rcDNA in the nucleus (∼20%) was only modestly higher than in the cytoplasm. If stably encapsidated nuclear rcDNA is a precursor to cccDNA (a), uncoating is the only slow step. If stable intranuclear capsids are a dead-end product nuclear import is rate-limiting. (**B**) HBV. Nuclei contained ≤10% MN resistant full-length rcDNA and <5% cccDNA; ≥80% of the rcDNA were MN sensitive. Hence conversion into these forms is efficient. About one third of the sensitive rcDNA could be precipitated with anti-HBc antibody; based on its genome region-selective sensitivity against MN we propose this fraction to represent partially opened capsids. About two thirds of the rcDNA was not precipitated, consistent with complete release. Hence initiation of uncoating is efficient but complete uncoating appears to be slow. Roughly two thirds in either fraction were protein-free, indicating that ∼40% of the nuclear HBV rcDNA were completely uncoated and no more linked to intact polymerase. This implies a major block for HBV in a subsequent step towards cccDNA which does not operate for DHBV. Little if any evidence was found in support of pathway b which implies partial uncoating and polymerase removal to occur in the cytoplasm.

Formation and amplification of cccDNA is essential for the establishment and maintenance of HBV infection. The underlying molecular pathway is poorly understood, not the least because HBV in transfected human hepatoma cell lines, despite efficient replication, produces very little cccDNA even if surface protein expression is prevented. Unexpectedly, our cross species transfection experiments revealed that the very same cell lines support efficient conversion of DHBV rcDNA into cccDNA. Our characterization of the intranuclearly accumulating rcDNA species showed that initiation of nuclear uncoating of the HBV rcDNA was highly efficient whereas complete release from the capsid and complete removal of the covalently linked polymerase contribute to limiting cccDNA formation. However, ∼40% of the nuclear rcDNA were apparently fully capsid-released and polymerase-free, hence a major block lies in the actual rcDNA to cccDNA conversion process; for DHBV, such a block does not appear to exist (Figure 10).

### Validity of data

The conclusion of efficient DHBV but poor HBV rcDNA to cccDNA conversion is based on the relative intensities of Southern blot signals at the rcDNA and cccDNA positions in nuclear preparations; a ratio of about 1∶1 for DHBV but 10–20∶1 for HBV was observed in more than 20 independent preparations. The selective partial resistance against Apa LI digestion of nuclear HBV rcDNA (Figure 3, Figure S5) strongly suggests that excessive nicking of HBV (but not DHBV) cccDNA is not a major cause for the strong rcDNA versus weak cccDNA signals, consistent with data from stably transfected cells [12], [13]. Another explanation would be that cccDNA of HBV has a much shorter half-life than that of DHBV. The in vivo half-life of cccDNA may be in the order of weeks to months [11], [34], [35] but the rates of synthesis versus degradation are subject to numerous factors, including cell division and immune responses. Closest to our setting are values derived from inducible cell lines in which rcDNA resynthesis was blocked by nucleoside analogs, with estimates of >48 h for DHBV [36] and >10 to 24 d for HBV [37]. Both values are long compared to the time frame of our experiments and, if anything, HBV cccDNA seems to be more stable than DHBV cccDNA. Hence it appears justified to assume that the weak HBV cccDNA signals truly reflect a slow rcDNA to cccDNA conversion rate.

### What limits the rate of HBV cccDNA formation?

Formation of cccDNA must involve nuclear transport of the rcDNA, its release from the capsid and removal of the bound polymerase to allow for the subsequent generation of precisely unit-length plus-strand and minus-strand DNA and ligation of their ends.

Regarding transport, the fraction of nuclear versus total rcDNA was about 2-fold higher for DHBV than for HBV (Figure 4); assuming that one cccDNA molecule originates from one imported rcDNA molecule, and given the about equal amounts of both species in DHBV transfected nuclei (Figure 2, 4) this ratio increases to 4∶1. Hence a lower import efficiency of HBV rcDNA can explain only part of the lower cccDNA accumulation.

Irrespective of mechanistic details (see below), the imported rcDNA molecules must eventually be released from the capsid to become accessible for the late repair steps in cccDNA formation. Nearly all of the nuclear, yet little of the cytoplasmic, full-length HBV rcDNA was sensitive to MN (Figure 4, 5, 6), and thus no more protected by an intact capsid shell; hence initiation of uncoating was highly efficient. Curiously though, MN treatment did not induce complete rcDNA degradation; instead, species with intermediate electrophoretic mobility accumulated, suggesting the existence of a distinct uncoating intermediate (see below). Nuclear DHBV rcDNA, in contrast, was as resistant against MN as cytoplasmic rcDNA, implying genome release for DHBV but not for HBV as potentially overall rate-limiting in cccDNA formation.

### Complete versus partial release from the capsid of nuclear HBV rcDNA as one step limiting cccDNA formation

Nuclear (save cccDNA) and cytoplasmic DNAs of both viruses occurred in core protein associated form (Figure 5). DNA of either virus immunoprecipitated from the cytoplasm was largely resistent against MN, consistent with stable encapsidation. For DHBV, this also held for the nuclear immunopellet whereas drastic differences between cytoplasmic and nuclear fractions were revealed for HBV. The anti-HBc antibody (a monoclonal antibody recognizing a linear epitope exposed on intact capsids yet also on denatured core protein; [38]) precipitated full-length rcDNA from either fraction, yet selectively the nuclear full-length rcDNA was highly sensitive to MN, though not over its entire length. Hence a fraction of the HBV rcDNA in the nucleus was still associated with core protein and this association was likely responsible for partial nuclease protection. Together, these data indicate that the HBV nucleocapsid structure is drastically altered upon nuclear import such that the packaged rcDNA is at least partially exposed; the surprisingly distinct nature of the resulting rcDNA - core protein complexes is discussed below.

Failure of all nuclear HBV rcDNA molecules to be completely released from core protein could have represented the major rate-limiting step in cccDNA formation. However, about two thirds of the nuclear full-length rcDNA could not be immunoprecipitated (Figure 9) although the same amount of antibody precipitated ≥95% of the much more abundant cytoplasmic rcDNA. Hence partial as opposed to complete capsid release contributes to poor HBV rcDNA to cccDNA conversion.

### Polymerase protein removal from HBV rcDNA is not overall rate-limiting

Cytoplasmic DNA of either virus not treated with PK generated signals that were less than 10% as intense as those obtained after artificial deproteinization (Figure 7, 8, S5, S7); even lower values were reported by Gao and Hu [12]. We can not exclude that these apparently protein-free DNAs are present in genuinely cytoplasmic nucleocapsids [26] but they could as well arise from molecules that during work-up have been subject to fortuitous proteolysis or leakage from ruptured nuclei, as supported by the inability of virion-derived nucleocapsids to generate any significant amount of protein-free rcDNA (see below). Very obvious was, in contrast, the high proportion of protein-free HBV rcDNA in the purified nuclei. Consistently about two thirds were recovered without PK treatment from various samples (Figure 7, 8, S5). The low abundance of nuclear HBV rcDNA prevented detection of significant differences in the polymerase-linkage status of core protein associated versus free rcDNA. We therefore currently assume that a similar proportion of rcDNA molecules lacking intact polymerase is present in either fraction (Figure 10B). Even then, about 40% of the nuclear rcDNA were both “protein-free” and completely released from the capsid. If the individual nuclear species are true precursors of one another, a major block in HBV cccDNA formation must occur further downstream. Given the operational definition of “protein-free”, this could involve removal of amino acid remnants from the polymerase from the minus-strand 5′ end, yet also (a) subsequent step(s). Defining the chemical nature of the DNA ends in the protein-free species therefore remains a major objective.

For DHBV, the simple model outlined as pathway b (Figure 10A) may be valid; however, the stable encapsidation and low degree of deproteinization (∼20%) of the nuclear rcDNA (Figure 4B, 5, 7) versus high cccDNA content are also compatible with the existence of two kinds of capsids; one from which rcDNA is rapidly released and efficiently converted into cccDNA such that no stable intermediates accumulate (Figure 10A, pathway b), and another in which uncoating is blocked or slowed down, represented by the nuclease resistant rcDNA (Figure 10A, pathway a). Kinetic studies will be required for a distinction. Remarkably though, already 1990 Summers and coworkers [20] observed an accumulation of apparently protein-free rcDNA in primary duck hepatocytes infected with pseudotyped surface protein-deficient DHBV and suspected this might represent an immediate precursor to cccDNA.

### Nuclear import-mediated disassembly appears to be initiated at specific sites of the HBV nucleocapsid

The exact nature of the partially nuclease resistant HBV rcDNA - core protein complexes in the nucleus is not yet clear; the status of the core protein may further be probed using assembly status dependent antibodies (for review: [39]). Conversely, however, our characterization of the DNA in these complexes revealed several unexpected aspects. First, although MN has non-sequence specific endo- and exonuclease activity, MN treatment generated a reproducible pattern (Figure 4, 7) of faster migrating DNAs, with a sharp upper boundary at a position where double-stranded DNA of about 2.7 kb would migrate. These species did not represent stably encapsidated, splicing-derived shorter DNAs [32], [33], [40] but rather double-stranded linear molecules lacking ∼500 bp of viral genome sequence. Most surprisingly, digestion with four different restriction enzymes produced distinct fragments which is only compatible with MN digestion of a defined genome region relative to these restriction sites; accordingly, the MN sensitive region is roughly bordered by the start of the minus-strand DNA and the end of the core protein ORF (Figure 6). Indeed, the fifth restriction enzyme, Bsp EI, whose single recognition site locates to this region, did not detectably alter mobility of the MN treated DNA although it completely linearized rcDNA. These data strongly suggest that nuclear disassembly of the HBV nucleocapsid shell initiates at specific sites defined by their relative position to the packaged genome, and that these partially opened nucleocapsids may represent a novel, transiently stable uncoating intermediate.

### Implications for the mechanism of hepadnaviral genome uncoating and deproteinization

Conceivable mechanisms for polymerase removal include nucleolytic cleavage of a piece of rcDNA which carries the protein, or specific cleavage of the Tyr-DNA-phosphodiester linkage; both mechanisms, possibly coupled to proteolysis, have been described for repair of cellular protein-DNA adducts [41]; our unpublished data (C. Königer, M. Nassal, J. Beck) indicate that certain Tyrosyl-DNA phosphodiesterases are indeed able to specifically cleave the polymerase from the DNA in vitro. An alternative model proposes that nucleocapsids themselves harbor an intrinsic ability for polymerase removal, perhaps via capsid-associated cellular factors, such that deproteinization as well as partial capsid opening precede nuclear import [13], [26]. Evidences were the reported presence of substantial amounts of protein-free and partially nuclease sensitive rcDNA in the cytoplasm, and the formation of small amounts of protein-free DNA in DHBV nucleocapsids derived from serum virions upon prolonged EPR conditions.

Our results do not support this view. First, in line with results from others [12] we found only a small fraction of the cytoplasmic (but most of the nuclear) HBV rcDNA in protein-free form. Second, in our hands the cytoplasmic HBV rcDNA was largely nuclease resistant, whereas most of the nuclear rcDNA was nuclease sensitive (Figure 4, 5). Third, we were unable to detect protein-free rcDNA in serum-derived HBV nucleocapsids upon prolonged EPR although the endogenous polymerase was clearly active (Figure 9). For DHBV, the same conditions led indeed to small amounts of protein-free DNA, as reported [26]. However, protein removal was extremely inefficient (<0.3%), and it concerned exclusively dlDNA, not rcDNA (Figure 9); notably, this was also observed by the authors when they analyzed cytoplasmic rather than virion-derived nucleocapsids [26]. While dlDNA can be circularized by nonhomologous end joining [42], most of the resulting molecules carry insertions or deletions, preventing generation of functional progeny virus. Thus capsid-autonomous deproteinization of dlDNA may exist but does not appear to reflect a major pathway in cccDNA synthesis.

The exclusive presence and strong enrichment, respectivley, of partially nuclease sensitive and protein-free HBV rcDNA in the nucleus in our experiments (Figure 4, 5) strongly favors that uncoating and polymerase removal are dependent on nuclear import. Furthermore, the polymerase linkage of the core protein associated MN products (Figure 7) yet overall large fraction of protein-free rcDNA in the nucleus (Figure 7, 8) suggests that uncoating precedes polymerase removal. Altogether, our data are much more compatible with a nuclear import-dependent, cell factor-mediated generation of protein-free rcDNA (Figure 10) than with a capsid-autonomous process in the cytoplasm. This would be in line with nuclear important-mediated alterations in capsid properties seen in digitonin-permeabilized cells [25]. Moreover, continued inhibition of viral polymerase activity during HBV infection of primary tupaia hepatocytes [30] and of HepaRG cells [43], the as yet only HBV-infectable human cell line [44], did not prevent cccDNA formation, further supporting a dominant role for cellular factors.

The reasons for the partially discordant results are not obvious; cell type or cell clone specific differences are not excluded. However, while we were able to prepare nuclei essentially free from cytoplasmic contaminants, the reverse was not true. A more substantial nuclear contamination of the cytoplasmic fractions employed by Guo et al. would as well account for many of the seeming discrepancies.

### What are the determinants for virus specific regulation?

The efficient formation of DHBV cccDNA in HepG2 and Huh7 cells demonstrates that also in these cells the underlying pathways are principally operating; this may also hold for human cells of non-hepatic origin, as discussed in a previous publication [12]. Given the similar structures of HBV and DHBV rcDNA it is not obvious as to why conversion into cccDNA would be so much more efficient for DHBV. One option was that the plus-strand DNA in HBV capsids is less extended in comparison to DHBV capsids; however, HBV rcDNA in transfection-derived capsids from HepG2 cells was as mature as DHBV rcDNA (Figure 9). Another option is that the cellular repair activities operate in a sequence-specific manner because the two viruses share less than 40% sequence identity on the genome level [45]. Such sequence-dependence remains to be explored.

Perhaps most conceivable is that viral proteins differentially affect cccDNA formation rates, either directly or in concert with cellular factors. Absence of the surface proteins favored nuclear import for both viruses, yet with accumulation of cccDNA for DHBV, and of rcDNA for HBV. Hence more complex consequences of surface protein - core protein interactions, or an effect of the surface proteins on cellular events affecting cccDNA formation are not excluded. Nuclear import of rcDNA is mediated by the viral core proteins [24]; although the DHBV genome is even smaller than that of HBV its core protein is much larger, including a surface-exposed domain of unknown function and a differently organized C terminal nucleic acid binding domain [46]. Obviously, the rates of nuclear transport and the fates of the intranuclear capsids differed between the two viruses. Even after uncoating and polymerase removal, both proteins, or fragments thereof, in the nucleus could affect further rcDNA processing. Phosphorylation - dephosphorylation events in the core protein nucleic acid binding domains are important during replication of both viruses [33], [47], [48] but potentially differing consequences for cccDNA have not yet been addressed. Not the least, mammalian but not avian hepatitis B viruses encode an additional gene product, HBx, whose function is still elusive [49]; however, the ortholog from woodchuck hepatitis virus (WHV) appears to be crucial for in vivo infection in the natural host [50], [51]. If HBx was involved in cccDNA formation, its improper expression or functioning in the human hepatoma cells might limit HBV cccDNA formation.

In a preliminary attempt to reveal potential regulatory activities of gene products from one virus on cccDNA production by the other we co-transfected HepG2 cells with the surface-deficient variants of both viruses. HBV had little effect on nuclear DHBV rcDNA and cccDNA formation; vice versa, HBV replication appeared slightly reduced overall, with no enhancement of cccDNA accumulation. In view of the multiple options for positive and negative regulation this may not be too surprising. A more revealing approach will therefore be coexpression of one virus with defined individual gene products from the other.

### Final conclusions

Our study identified several steps downstream of initiation of nuclear uncoating that slow down (but do not completely prevent) HBV rcDNA to cccDNA conversion in human hepatoma cell lines. Because the same cells supported DHBV cccDNA formation very well, they can not completely lack the necessary molecular means. One option for the seeming discrepancy to efficient cccDNA formation during HBV infection in vivo is that cellular activities promoting the conversion are limited in these cell lines, another that inhibitory factors are overexpressed; this seems unlikely, though, because HBV cccDNA accumulation was not boosted in the avian LMH cells. Still another option is that rcDNA to cccDNA conversion is intrinsically slower for HBV than for DHBV. Determining cccDNA copy numbers in HBV infected human liver suffers from various experimental restrictions (see Introduction). Hence more telling than the final yields of cccDNA molecules per hepatocyte may be a comparison of the rates of cccDNA accumulation in HBV versus DHBV infection. In experimentally HBV infected chimpanzees, cccDNA levels during the early phase increased exponentially with a doubling time of around 4 days, expectedly paralleled by a corresponding increase in infected hepatocytes [11]; in ducks, the doubling time was only around 16 h [52]. These in vivo data would be compatible with DHBV cccDNA formation occurring at a higher rate yet at this time, a direct correlation with our findings remains speculative. Practically, however, the accumulation in the hepatoma cells of distinct nuclear HBV rcDNA forms that are likely intermediates in the rcDNA to cccDNA pathway will allow to much more thoroughly dissect the underlying molecular mechanisms. Efficient DHBV cccDNA formation in the same cells, on the other hand, provides a unique new tool to facilitate identification, e.g. by RNAi technology, of the human cellular factors involved in cccDNA biosynthesis and the screening for specific inhibitors that directly address the form of viral genome which ensures establishment and persistence of HBV infection.

## Materials and Methods

Detailed descriptions of the plasmid constructs and procedures employed are reported in Text S1.

### Plasmid constructs

Virus vectors contained 1.5× genome length sequences of HBV (genotype D, subtype ayw; Genbank accession no.: V01460) or DHBV (DHBV16; accession no.: K01834). Numbering for HBV starts with the first nucleotide of the core open reading frame [53], for DHBV with the last nucleotide of the unique Eco RI site [54]. Plasmid pCH-9/3091 [55] contains a 1.05× genome of the same HBV isolate under control of the cytomegalovirus immediate early (CMV-IE) promoter. Surface protein deficient HBV carried mutations at positions 1399 and 1438, introducing a stop codon in the preS2 ORF and a Met>Thr exchange at the S ORF start. In surface deficient DHBV, a G>A exchange at position 1165 creates a stop codon in the S ORF [56]. In the splicing-deficient HBV construct, A1769 in the major splice acceptor consensus site CAG|G (A1769 underlined) was changed to C.

### Cell culture and transfection

HepG2, Huh7 and LMH cells were cultured essentially as previously described [18], [33]. Transfections were performed using TransIT-LT1 reagent as recommended by the manufacturer (Mirus). Cells were harvested 3 days post transfection, unless indicated otherwise.

### Extraction and detection of viral DNA

Briefly, cells were lysed NP40 lysis buffer, and the nuclei were separated by low speed centrifugation [33], [57]. Alternatively, nuclei were purified by sucrose gradient sedimentation (see below). Total cell extracts were obtained by lysis in 0.5% SDS. After different treatments, including or not including incubation with proteinase K (PK) and/or micrococcus nuclease (MN), viral DNAs were isolated using QIAamp silica columns (Qiagen), or by conventional phenol extraction as indicated. Dpn I (NEB) and Plasmid-Safe DNase (Epicentre Biotechnologies) were applied as suggested by the manufacturers. Viral DNAs on Southern blots were visualized using ^32^P labeled full-length genome DNA probes.

### Isolation of cell nuclei by sucrose gradient sedimentation

Cells were subjected to detergent lysis and subsequent sucrose step gradient centrifugation, essentially as described [29].

### Cross-contamination analysis of gradient purified nuclei

Absence of cytoplasmic contamination from the purified nuclei was assessed by comparing the presence of cytoplasmic poly-A binding protein (PABP) and nuclear histone H3 in total versus nuclear extracts. Nonspecific association of viral capsids with nuclei was addressed by mixing capsid-containing cytoplasmic extracts from transfected cells with total lysates from untransfected cells, followed by sucrose gradient purification of the nuclei. Permeability of the isolated nuclei for exogenously added MN was confirmed by fragmentation of the chromosomal DNA.

### Distinction between nuclear protein-free rcDNA and nicked cccDNA

Identity of cccDNA was confirmed by linearization upon incubation with single-cutter restriction enzymes and resistance against heat denaturation. RC-DNA and nicked cccDNA were distinguished by the defined versus randomly positioned discontinuites in the DNA strands. The unique recognition site for Apa LI is located immediately upstream of the plus-strand 5′ end in RC-DNA; plus-strands not extended through this region prevent cleavage of RC-DNA whereas randomly nicked cccDNA is not affected. Cleavability at sites further upstream (Nco I) or downstream (Fsp I) served as control.

### Immunoprecipitation of viral nucleocapsids

For immunoprecipitation (IP) of core protein associated DNAs monoclonal antibody 312 against HBV core protein [38] and a polyclonal antiserum raised against recombinant DHBV capsids [58] were used. For IPs from purified nuclei, the nuclei were incubated in 0.75× radioimmunoprecipitation (RIPA) buffer (1× RIPA buffer is 20 mM Tris (pH 7.2), 1% sodium deoxycholate, 1% Triton X-100, 0.1% sodium dodecyl sulfate, 150 mM NaCl), and briefly sonicated; cytoplasmic extracts were adjusted to 0.75× RIPA as well. IPs were then performed as previously described [58], with the specific antibody immobilized to protein A or protein G Sepharose (GE Healthcare). Associated DNAs were isolated as described above.

### Endogenous polymerase reaction

Virion-derived nucleocapsids were obtained from highly viremic sera by sedimentation in Nycodenz gradients containing 0.5% (v/v) NP-40, and subsequently incubated in EPR buffer containing 1 mM each of the four dNTPs for 16 h, as described [26].

### Quantitation and statistical evaluation

Southern blot signal intensities were determined by phosphorimaging, using AIDA (Fuji) or ImageQuant software (GE Healthcare). Background corrections were performed by subtracting from the signal of interest the value obtained for an equally sized area in the same lane. Quantitations from each blot were performed in duplicate. In several cases, dilution series were used to more accurately determine relative amounts of a given species in different compartments or after different treatments. Standard deviations were derived from at least two and mostly more independent experiments and calculated using Microsoft Excel software. Statistical significance was evaluated using Graphpad Prism 5 for Mac software.

## Supporting Information

Figure S1 **(A)** Schematic view of hepadnavirus intracellular recycling. Upon infection, encapsidated polymerase-linked rcDNA is transported to the nucleus and eventually converted into cccDNA. This requires release from the capsid shell (uncoating) and detachment from the covalently bound polymerase, plus further steps outlined in (B). The cccDNA episome serves as template for all viral transcripts including pgRNA. Via interaction with the polymerase, pgRNA is packaged into viral capsids and reverse transcribed into ssDNA of minus polarity. Due to the protein-priming mechanism, the 5′ end of the minus DNA is covalently linked to the polymerase. Synthesis of the plus strand, primed by an RNA oligonucleotide (dashed red extension in B) derived from the pgRNA 5′ end, yields new encapsidated rcDNA which upon interaction of the nucleocapsid with the viral surface proteins can be secreted in enveloped virions. Alternatively, and particularly in the absence of the surface proteins, nuclear import and cccDNA formation may occur again (intracellular recycling). The dashed lines indicate poorly understood parts of the cycle. RNA is shown in red, DNA in blue. **(B)** Multiple steps required for polymerase-linked rcDNA to cccDNA conversion. Before the final ligation to cccDNA, the protein-bound rcDNA must undergo multiple processing steps to ensure formation of precisely unit-length plus and minus strand DNA with ligation-compatible ends.
(0.12 MB PDF)

Figure S2 DHBV versus HBV replication and cccDNA formation in human Huh7 cells. Huh7 cells were transfected with vectors encoding wild-type and surface-deficient DHBV or HBV. The experimental set-up was as in Figure 1 and 2. The Dpn I digestion in the nuclear DHBV samples was less than complete yet PsD removed most of the remaining plasmid DNA but not viral rcDNA and cccDNA. Note the similarly efficient cccDNA formation by the surface-deficient DHBV in Huh7 as in LMH (Figure 1) and HepG2 cells (Figure 2). For the nuclear HBV samples a five times longer exposure is shown to better reveal the weak signals.
(1.17 MB PDF)

Figure S3 **(A)** Kinetics of DHBV and HBV cccDNA formation in HepG2 cells. HepG2 cells were transfected with vectors for surface-deficient DHBV or HBV. Cells were harvested at the indicated day post transfection. Viral DNAs from the cytoplasm were extracted after MN and PK treatment; nuclear DNAs were prepared without MN but with PK treatment; the isolated nuclear DNAs were then treated with Dpn I plus PsD. Markers (M) were mixtures of 10 pg linear viral genomes plus 60 pg of circular plasmids, each carrying about 500 bp of the respective virus sequence. The panel on the right shows a four times longer exposure of the nuclear HBV samples. Note the comparable increases with time in nuclear rcDNA and cccDNA for both viruses; the virus-specific pattern of high nuclear cccDNA vs. rcDNA for DHBV and the reverse pattern for HBV did not change. **(B)** Increased transcription of HBV pgRNA does not increase cccDNA accumulation. HepG2 cells were transfected with vectors for surface-deficient HBV in which pgRNA transcription was either driven by the HBV core promoter (HBV) or the cytomegalovirus immediately early promoter (CMV). Cytoplasmic and nuclear viral DNAs were prepared as in Figure S3A. The indicated fractions of the total cytoplasmic and nuclear samples, obtained from one well of a 6-well plate, were loaded. The CMV promoter controlled vector increased the levels of replicative intermediates about 3- to 4-fold but did not induce a detectable increase in cccDNA accumulation.
(1.64 MB PDF)

Figure S4 Validation of nuclei purification protocol. **(A)** Distribution of cytoplasmic poly-A binding protein (PABP) and nuclear histone H3. Cell nuclei (Nu) were prepared by sucrose gradient sedimentation as described in Text S1; from an aliquot of the same cells, a total extract (To) was prepared before separation. Samples were analyzed by Western blotting using antibodies against PABP (kindly provided by M. Hentze, EMBL Heidelberg, Germany) and histone H3 (Bethyl Laboratories) and chemiluminescent detection. Positions of size marker proteins are indicated on the right; *, non-specifically crossreacting band. Note that the cytoplasmic PABP is exclusively detectable in the total lysate. **(B)** Cytoplasmic viral nucleocapsids do not detectably cosediment with nuclei. Cytoplasmic extracts (Cy) from HepG2 cells transfected with vectors for DHBV or HBV were mixed with total lysates from non-transfected cells. Subsequently, nuclei from the mixture were separated by the gradient sedimentation protocol. Viral DNAs were prepared from the cytoplasmic extracts and from the purified nuclei by micrococcal nuclease (MN) plus proteinase K (PK) treatment and analyzed by Southern blotting. Note the complete absence of viral DNA from the nuclei. **(C)** Isolated nuclei are permeable to exogenously added nuclease. Nuclei prepared by the gradient sedimentation protocol were incubated for 1 h at 37°C with the indicated amounts of micrococcal nuclease (MN). Total DNA was prepared and analyzed by agarose gel electrophoresis and ethidium bromide staining. The dose-dependent generation of bands of about 0.15 kbp and multiples thereof indicates cleavage between nucleosomes, as expected. Because a small amount of oligomers was still visible after 1 h treatment, a 5 h incubation time was used in experiments addressing nuclease resistance vs. sensitivity of nuclear viral DNAs.
(1.08 MB PDF)

Figure S5 Characteristics of nuclear viral DNAs. **(A)** Evidence that the band at the cccDNA position is truly cccDNA. Viral DNAs from purified nuclei of HepG2 cells transfected with vectors for surface-deficient DHBV or HBV were digested with Dpn I and PsD. Samples were loaded without further treatment (native), after prior incubation with Eco RI which has a single recognition site in either viral genome (Eco RI), or after heat denaturation (denat). Eco RI treament converted both bands into one new band migrating at the position of double-stranded linear (DL) DNA; heating converted the band at the rcDNA position into faster migrating single-stranded (SS) DNA whereas the band at the cccDNA position was not affected, as expected. The right panel shows a longer exposure of the center panel to reveal the weak HBV cccDNA signals. **(B)** Evidence that the band at the rcDNA position is not derived from extensive nicking of cccDNA. HepG2 cells were transfected with the vector for surface-deficient HBV. Viral DNAs were isolated from the cytoplasmic extract by treatment with MN plus PK, or from purified nuclei either with, or without prior PK treatment; PK treated DNA was further incubated with Dpn I but not PsD to preserve the plasmid DNA Dpn I fragments. Finally, the isolated DNAs were analyzed directly (ø), or after admixing 50 pg of a DHBV plasmid and incubation with Nco I, Apa LI, or Fsp I. MH, HBV marker fragments; MD, linear DHBV genome. Hybridization with an HBV specific probe (upper panels) confirmed (see Figure 3) that about one third of the rcDNA signal was resistant to cleavage by Apa LI but not by Nco I and Fsp I, and that this also held for nuclear protein-free (i.e. not PK treated) rcDNA. Activity of Apa LI was demonstrated by the absence of a Dpn I fragment containing internal restriction sites for Apa LI and Fsp I but not Nco I, and by the appearance of DHBV plasmid fragments of the expected sizes (*) upon rehybridization with a DHBV specific probe which also revealed MD (**). Due to their higher abundance, the cytoplasmic but not the nuclear HBV signals from the previous hybridization are still visible.
(3.88 MB PDF)

Figure S6 Most of the DHBV but little of the HBV full-length rcDNA in the nucleus is resistant to nuclease. To evaluate the intracellular distribution of nuclease resistant viral DNAs, HepG2 cells were transfected with vectors for surface-deficient DHBV (left panel) or HBV (right panel). DNA was extracted, after prior MN and PK treatment, from total cells or from gradient-purified nuclei. As for the samples shown in Figure 4A which instead had been treated with PK followed by Dpn I plus PsD, serially diluted samples were loaded on the gel. Loading volumes are indicated in percent of the total sample volume, obtained from one well of a 6-well plate. For DHBV, a comparable fraction of rcDNA was recovered from the nuclei upon either treatment (about one third; compare the signal intensities for the 30% nuclear versus 10% cytoplasmic aliquot), indicating the majority of nuclear rcDNA was stably encapsidated. For HBV, in contrast, virtually no signal for intact rcDNA was visible in the nuclear sample; instead, DNAs with an intermediate electrophoretic mobility accumulated. The fraction of nuclease-resistant versus total nuclear HBV rcDNA (∼10%) was estimated from experiments like that shown in Figure 4B.
(0.66 MB PDF)

Figure S7 Core protein association, nuclease resistance and polymerase linkage status of HBV rcDNA in the cytoplasm of transfected HepG2 cells. Immunoprecipitations were performed as in Figure 5; the relevant nuclear samples from this experiment are shown in the right panel of Figure 5B. Note that the IP was specific and efficient (high signal in the αHBc pellet vs. supernatant; low signal in the mock IP pellet vs. supernatant), and that most of the cytoplasmic rcDNA was resistant against nuclease (similarly strong signals with vs. without MN treatment) and still linked to polymerase (low signals without vs. with prior PK treatment). ø, cytoplasmic lysate directly treated with MN (no IP).
(6.64 MB PDF)

Text S1 This file contains a detailed description of experimental procedures not contained in the brief general Material and Methods section of the main text, plus a list of supporting references.
(0.08 MB DOC)
