# Supplementary figures and images for: Generation of Covalently Closed Circular DNA of Hepatitis B Viruses via Intracellular Recycling Is Regulated in a Virus Specific Manner
Source: PLoS Pathog. 2010 Sep 2;6(9):e1001082. doi: 10.1371/journal.ppat.1001082 (PMC2932716; doi:10.1371/journal.ppat.1001082)

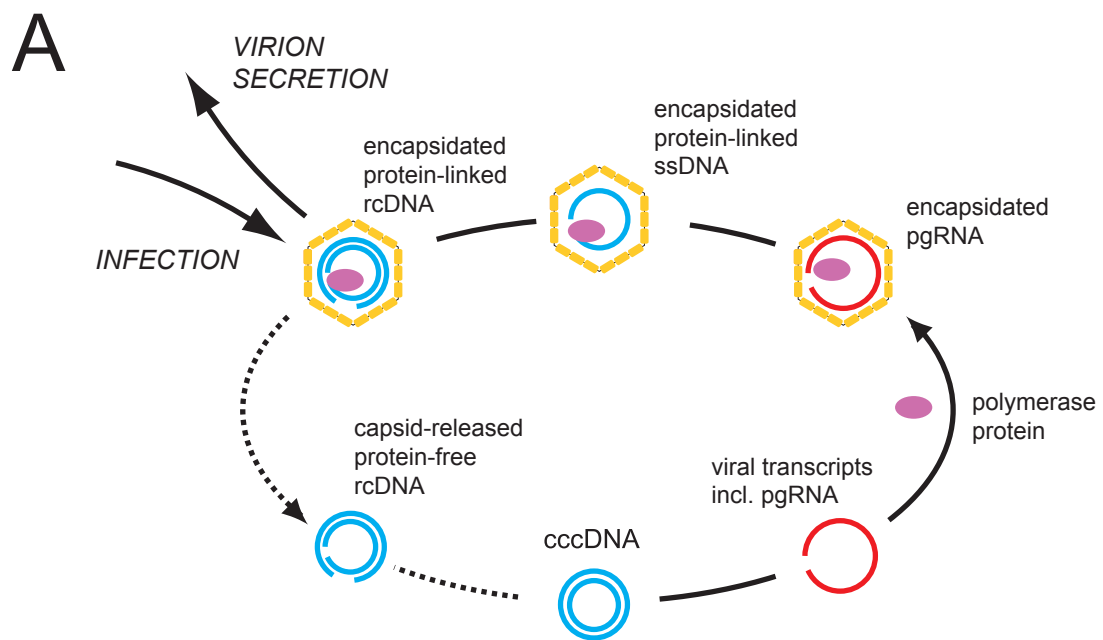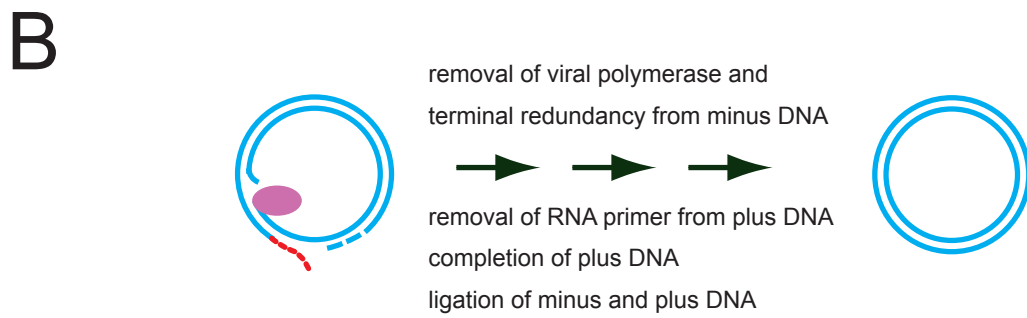

Supplement: Figure S1 — (A) Schematic view of hepadnavirus intracellular recycling. Upon infection, encapsidated polymerase-linked rcDNA is transported to the nucleus and eventually converted into cccDNA. This requires release from the capsid shell (uncoating) and detachment from the covalently bound polymerase, plus further steps outlined in (B). The cccDNA episome serves as template for all viral transcripts including pgRNA. Via interaction with the polymerase, pgRNA is packaged into viral capsids and reverse transcribed into ssDNA of minus polarity. Due to the protein-priming mechanism, the 5′ end of the minus DNA is covalently linked to the polymerase. Synthesis of the plus strand, primed by an RNA oligonucleotide (dashed red extension in B) derived from the pgRNA 5′ end, yields new encapsidated rcDNA which upon interaction of the nucleocapsid with the viral surface proteins can be secreted in enveloped virions. Alternatively, and particularly in the absence of the surface proteins, nuclear import and cccDNA formation may occur again (intracellular recycling). The dashed lines indicate poorly understood parts of the cycle. RNA is shown in red, DNA in blue. (B) Multiple steps required for polymerase-linked rcDNA to cccDNA conversion. Before the final ligation to cccDNA, the protein-bound rcDNA must undergo multiple processing steps to ensure formation of precisely unit-length plus and minus strand DNA with ligation-compatible ends. (0.12 MB PDF) [file ppat.1001082.s001.pdf]

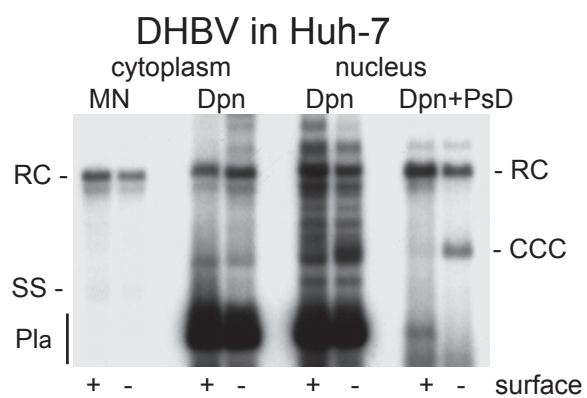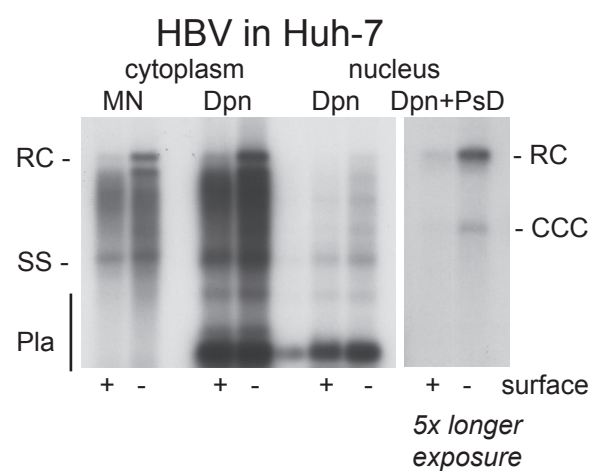

Supplement: Figure S2 — DHBV versus HBV replication and cccDNA formation in human Huh7 cells. Huh7 cells were transfected with vectors encoding wild-type and surface-deficient DHBV or HBV. The experimental set-up was as in Figure 1 and 2. The Dpn I digestion in the nuclear DHBV samples was less than complete yet PsD removed most of the remaining plasmid DNA but not viral rcDNA and cccDNA. Note the similarly efficient cccDNA formation by the surface-deficient DHBV in Huh7 as in LMH (Figure 1) and HepG2 cells (Figure 2). For the nuclear HBV samples a five times longer exposure is shown to better reveal the weak signals. (1.17 MB PDF) [file ppat.1001082.s002.pdf]

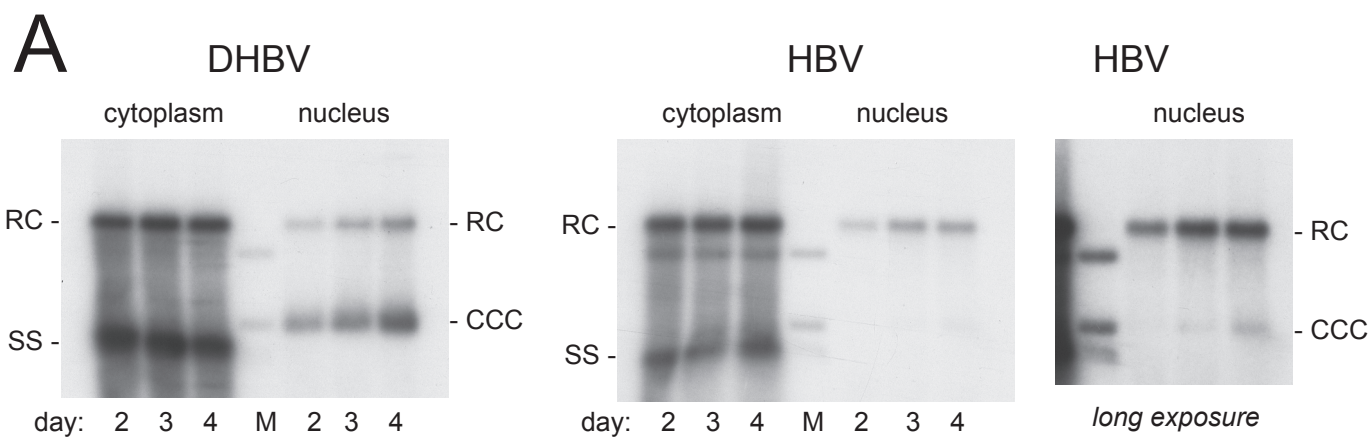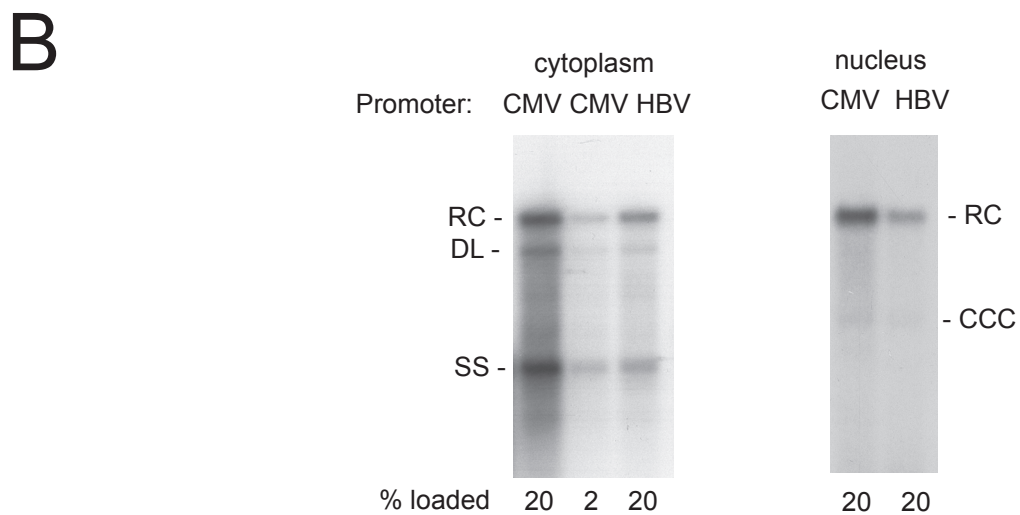

Supplement: Figure S3 — (A) Kinetics of DHBV and HBV cccDNA formation in HepG2 cells. HepG2 cells were transfected with vectors for surface-deficient DHBV or HBV. Cells were harvested at the indicated day post transfection. Viral DNAs from the cytoplasm were extracted after MN and PK treatment; nuclear DNAs were prepared without MN but with PK treatment; the isolated nuclear DNAs were then treated with Dpn I plus PsD. Markers (M) were mixtures of 10 pg linear viral genomes plus 60 pg of circular plasmids, each carrying about 500 bp of the respective virus sequence. The panel on the right shows a four times longer exposure of the nuclear HBV samples. Note the comparable increases with time in nuclear rcDNA and cccDNA for both viruses; the virus-specific pattern of high nuclear cccDNA vs. rcDNA for DHBV and the reverse pattern for HBV did not change. (B) Increased transcription of HBV pgRNA does not increase cccDNA accumulation. HepG2 cells were transfected with vectors for surface-deficient HBV in which pgRNA transcription was either driven by the HBV core promoter (HBV) or the cytomegalovirus immediately early promoter (CMV). Cytoplasmic and nuclear viral DNAs were prepared as in Figure S3A. The indicated fractions of the total cytoplasmic and nuclear samples, obtained from one well of a 6-well plate, were loaded. The CMV promoter controlled vector increased the levels of replicative intermediates about 3- to 4-fold but did not induce a detectable increase in cccDNA accumulation. (1.64 MB PDF) [file ppat.1001082.s003.pdf]

**A**

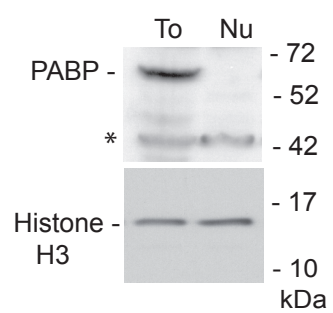

**B**

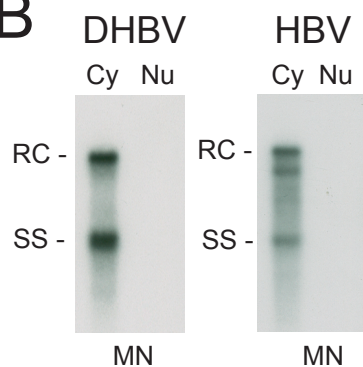

**C**

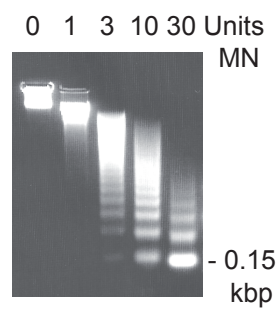

Supplement: Figure S4 — Validation of nuclei purification protocol. (A) Distribution of cytoplasmic poly-A binding protein (PABP) and nuclear histone H3. Cell nuclei (Nu) were prepared by sucrose gradient sedimentation as described in Text S1; from an aliquot of the same cells, a total extract (To) was prepared before separation. Samples were analyzed by Western blotting using antibodies against PABP (kindly provided by M. Hentze, EMBL Heidelberg, Germany) and histone H3 (Bethyl Laboratories) and chemiluminescent detection. Positions of size marker proteins are indicated on the right; *, non-specifically crossreacting band. Note that the cytoplasmic PABP is exclusively detectable in the total lysate. (B) Cytoplasmic viral nucleocapsids do not detectably cosediment with nuclei. Cytoplasmic extracts (Cy) from HepG2 cells transfected with vectors for DHBV or HBV were mixed with total lysates from non-transfected cells. Subsequently, nuclei from the mixture were separated by the gradient sedimentation protocol. Viral DNAs were prepared from the cytoplasmic extracts and from the purified nuclei by micrococcal nuclease (MN) plus proteinase K (PK) treatment and analyzed by Southern blotting. Note the complete absence of viral DNA from the nuclei. (C) Isolated nuclei are permeable to exogenously added nuclease. Nuclei prepared by the gradient sedimentation protocol were incubated for 1 h at 37°C with the indicated amounts of micrococcal nuclease (MN). Total DNA was prepared and analyzed by agarose gel electrophoresis and ethidium bromide staining. The dose-dependent generation of bands of about 0.15 kbp and multiples thereof indicates cleavage between nucleosomes, as expected. Because a small amount of oligomers was still visible after 1 h treatment, a 5 h incubation time was used in experiments addressing nuclease resistance vs. sensitivity of nuclear viral DNAs. (1.08 MB PDF) [file ppat.1001082.s004.pdf]

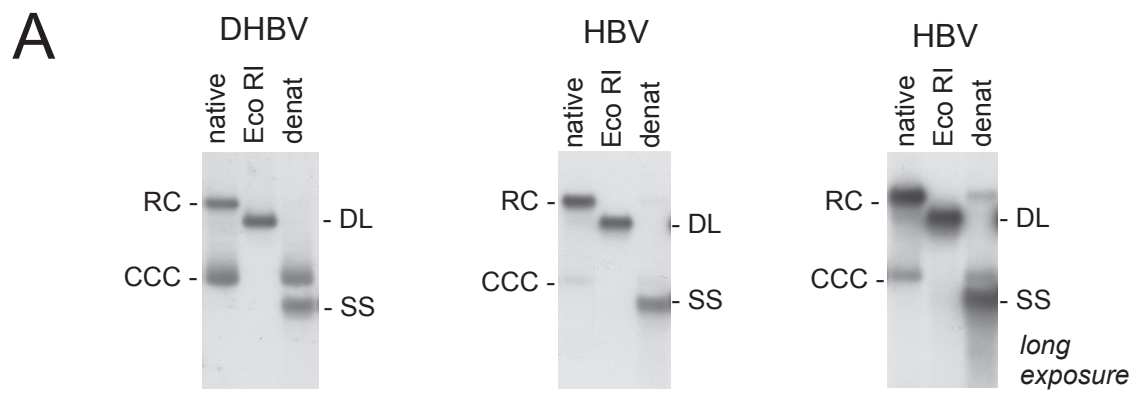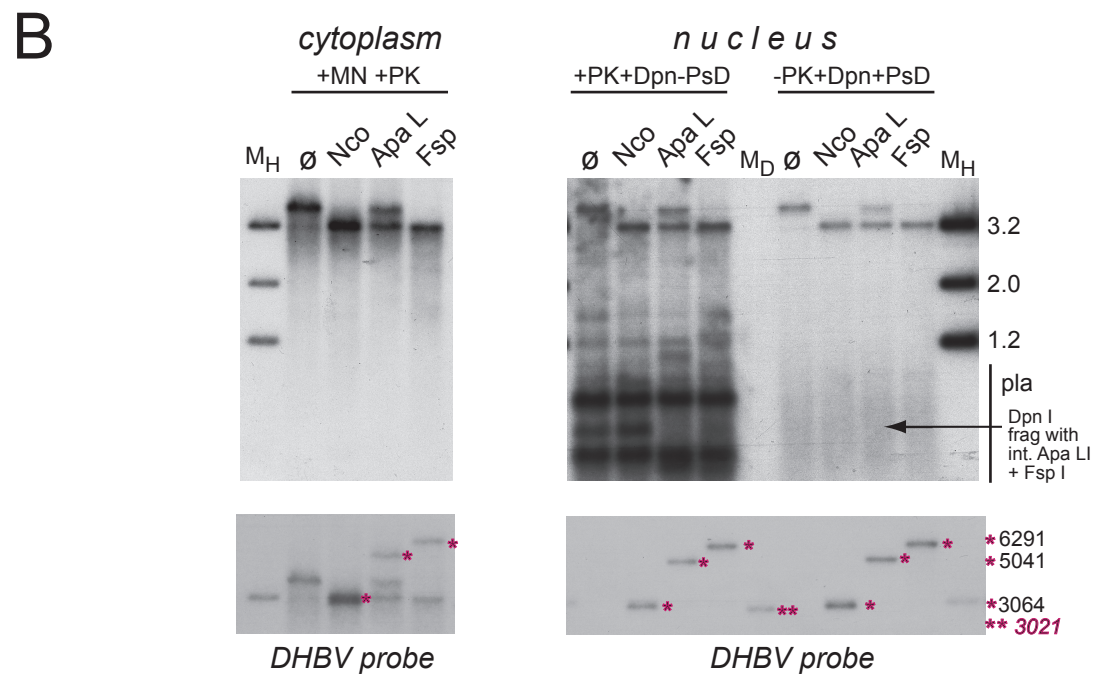

Supplement: Figure S5 — Characteristics of nuclear viral DNAs. (A) Evidence that the band at the cccDNA position is truly cccDNA. Viral DNAs from purified nuclei of HepG2 cells transfected with vectors for surface-deficient DHBV or HBV were digested with Dpn I and PsD. Samples were loaded without further treatment (native), after prior incubation with Eco RI which has a single recognition site in either viral genome (Eco RI), or after heat denaturation (denat). Eco RI treament converted both bands into one new band migrating at the position of double-stranded linear (DL) DNA; heating converted the band at the rcDNA position into faster migrating single-stranded (SS) DNA whereas the band at the cccDNA position was not affected, as expected. The right panel shows a longer exposure of the center panel to reveal the weak HBV cccDNA signals. (B) Evidence that the band at the rcDNA position is not derived from extensive nicking of cccDNA. HepG2 cells were transfected with the vector for surface-deficient HBV. Viral DNAs were isolated from the cytoplasmic extract by treatment with MN plus PK, or from purified nuclei either with, or without prior PK treatment; PK treated DNA was further incubated with Dpn I but not PsD to preserve the plasmid DNA Dpn I fragments. Finally, the isolated DNAs were analyzed directly (ø), or after admixing 50 pg of a DHBV plasmid and incubation with Nco I, Apa LI, or Fsp I. MH, HBV marker fragments; MD, linear DHBV genome. Hybridization with an HBV specific probe (upper panels) confirmed (see Figure 3) that about one third of the rcDNA signal was resistant to cleavage by Apa LI but not by Nco I and Fsp I, and that this also held for nuclear protein-free (i.e. not PK treated) rcDNA. Activity of Apa LI was demonstrated by the absence of a Dpn I fragment containing internal restriction sites for Apa LI and Fsp I but not Nco I, and by the appearance of DHBV plasmid fragments of the expected sizes (*) upon rehybridization with a DHBV specific probe which also revealed MD (* [file ppat.1001082.s005.pdf]

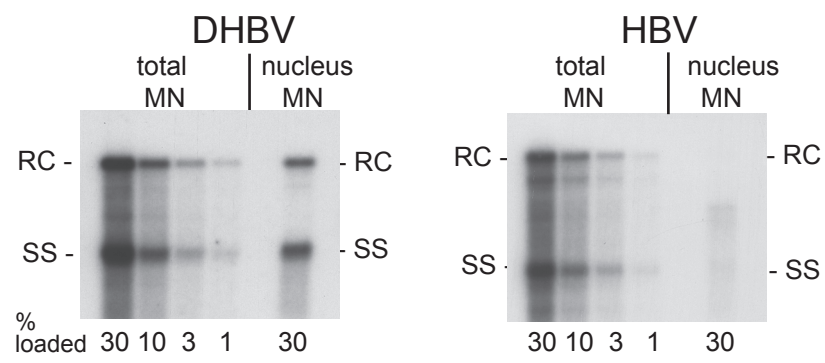

Supplement: Figure S6 — Most of the DHBV but little of the HBV full-length rcDNA in the nucleus is resistant to nuclease. To evaluate the intracellular distribution of nuclease resistant viral DNAs, HepG2 cells were transfected with vectors for surface-deficient DHBV (left panel) or HBV (right panel). DNA was extracted, after prior MN and PK treatment, from total cells or from gradient-purified nuclei. As for the samples shown in Figure 4A which instead had been treated with PK followed by Dpn I plus PsD, serially diluted samples were loaded on the gel. Loading volumes are indicated in percent of the total sample volume, obtained from one well of a 6-well plate. For DHBV, a comparable fraction of rcDNA was recovered from the nuclei upon either treatment (about one third; compare the signal intensities for the 30% nuclear versus 10% cytoplasmic aliquot), indicating the majority of nuclear rcDNA was stably encapsidated. For HBV, in contrast, virtually no signal for intact rcDNA was visible in the nuclear sample; instead, DNAs with an intermediate electrophoretic mobility accumulated. The fraction of nuclease-resistant versus total nuclear HBV rcDNA (∼10%) was estimated from experiments like that shown in Figure 4B. (0.66 MB PDF) [file ppat.1001082.s006.pdf]

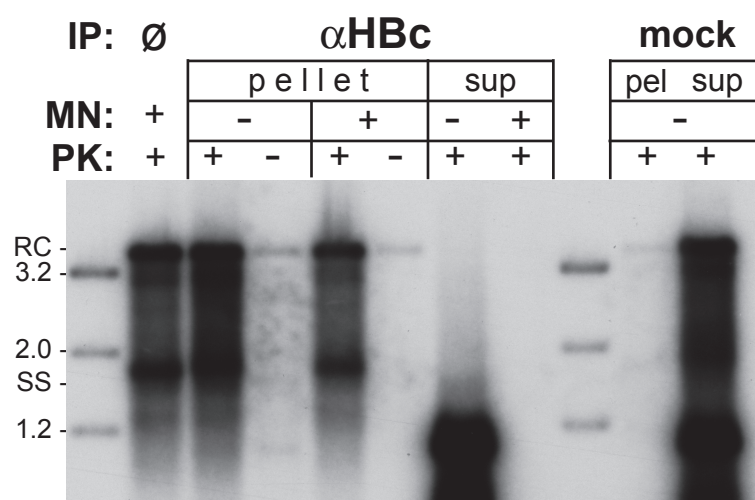

Supplement: Figure S7 — Core protein association, nuclease resistance and polymerase linkage status of HBV rcDNA in the cytoplasm of transfected HepG2 cells. Immunoprecipitations were performed as in Figure 5; the relevant nuclear samples from this experiment are shown in the right panel of Figure 5B. Note that the IP was specific and efficient (high signal in the αHBc pellet vs. supernatant; low signal in the mock IP pellet vs. supernatant), and that most of the cytoplasmic rcDNA was resistant against nuclease (similarly strong signals with vs. without MN treatment) and still linked to polymerase (low signals without vs. with prior PK treatment). ø, cytoplasmic lysate directly treated with MN (no IP). (6.64 MB PDF) [file ppat.1001082.s007.pdf]
