# Supplementary material for: Generation of Covalently Closed Circular DNA of Hepatitis B Viruses via Intracellular Recycling Is Regulated in a Virus Specific Manner
Source: PLoS Pathog. 2010 Sep 2;6(9):e1001082. doi: 10.1371/journal.ppat.1001082 (PMC2932716; doi:10.1371/journal.ppat.1001082)
Supplement: Text S1 — This file contains a detailed description of experimental procedures not contained in the brief general Material and Methods section of the main text, plus a list of supporting references. (0.08 MB DOC) [file ppat.1001082.s008.doc]

**Text S1.**

This file contains a detailed description of experimental procedures not contained in the brief general Materials and Methods section of the main text, plus a list of supporting references.

**Plasmid constructs**

The HBV plasmids applied in this study harbor a 5' terminal redundancy ranging from the Nsi I site at position 2346 to the Bgl II site at position 84, enabling the synthesis of mRNAs for all viral proteins from the autologous promoters. The surface-deficient construct contains two mutations at genome position 1399 and 1438, introducing an artificial stop codon within the large and middle surface protein open reading frame (ORF) and a Met>Thr exchange abrogating translation of the small surface protein [1]. Neither mutation alters the overlappingly encoded polymerase protein sequence. The CMV promoter controlled HBV vector pCH-9/3091 was previously described [2]. In surface deficient DHBV, a G>A exchange at position 1165 creates a stop codon in the S ORF [3]. In the splicing-deficient HBV construct, A1769 in the major splice acceptor consensus site CAG|G (A1769 underlined; |, intron-exon border) was changed to C. Suppression of splicing was shown by the absence from the mutant but presence in wild-type HBV DNA of an additional about 1 kb shorter amplification product upon near genome length PCR.

The terminal redundancy in the DHBV constructs ranges from the Bam HI site at position 1658 to the Sph I site at position 2850 [4]. A stop codon in the DHBV surface coding region was introduced by a G>A exchange at position 1165 [3] which also does not affect the polymerase protein. All plasmids were grown in an *E.coli* strain (Top10) proficient for dam-methylation to enable digestion with restriction enzyme Dpn I.

**Cell culture and transfection**

Chicken LMH cells were cultured in Iscove's Modified Dulbecco's Medium (Gibco) supplemented with 10% fetal calf serum (FCS), 100 U/ml streptomycin and 100 µg/ml penicillin. Human HepG2 and HuH7 cells were kept in High Glucose Dulbecco's Modified Eagle's Medium (PAA Laboratories) supplemented with FCS and antibiotics as described above. Culture dishes were coated with collagen (BD Biosciences) prior to seeding of HepG2 cells to facilitate a more uniform cell distribution and growth. The absence of Mycoplasma infection was assured with a PCR test kit from AppliChem. Species identity of the LMH cell line was verified by amplification and partial sequencing of the mitochondrial 16S rRNA gene with primers 5´-TCCAACATCGAGGTCGTAAAC-3´ and 5´-GTACCGCAAGGGAAAGATGAA-3´. Short tandem repeat authentification of the HepG2 and HuH7 cell lines was performed at a commercial DNA analytic laboratory (www.dna-analytic.de).

Cells were transiently transfected with 6 µl of TransIT-LT1 transfection reagent (Mirus Bio) and 2 µg of plasmid DNA per well of a 6-well dish; correspondingly more reagent and DNA were used for 10 cm dishes. Three days post transfection, the cells were detached by trypsin treatment, resuspended in culture medium and collected by centrifugation.

**Extraction and detection of viral DNA**

Cell pellets were resuspended in 0.5 ml of ice-cold lysis buffer (140 mM NaCl, 1.5 mM MgCl_2_, 50 mM Tris-HCl [pH 8.0] and 0.5% Nonidet P-40). Nuclei were separated from the cytoplasmic lysate by centrifugation at 2,000 rpm for 5 min (Eppendorf table top centrifuge). The pelleted nuclei were dissolved in ATL lysis buffer (QIAamp DNA Mini Kit, Qiagen) and subsequently digested with proteinase K. The lysate was treated with RNase A and passed through a QIAshredder column (Qiagen) to reduce viscosity. DNA was purified using QIAamp silica columns and digested with 20 units of restriction enzyme Dpn I (NEB). An aliquot of the reaction was additionally treated with 10 units of Plasmid safe DNase (Epicentre Biotechnologies) in the presence of 1 mM ATP overnight at 37°C. Supernatants containing the cytoplasmic lysate were cleared of cell debris by additional centrifugation for 5 min at 14,000 rpm and subsequently digested with 30 units of micrococcal nuclease (MN; GE Healthcare) for 5 h at 37°C in the presence of 2 mM CaCl_2_. MN was inactivated by adding EDTA to a final concentration of 5 mM. Viral DNA was subsequently purified with QIAamp silica columns. Alternatively, DNA was extracted from cytoplasmic lysate without prior MN treatment but instead incubated, after preparation, with Dpn I. DNA samples were separated on 1.4% agarose gels, transferred onto Hybond-XL nylon membranes (GE Healthcare) and hybridized with ^32^P-labeled full-length HBV or DHBV probes in Roti-Hybri-Quick solution (Roth, Germany). Ten picogram of unit length viral genomes, excised from the corresponding plasmid vectors, and/or 60 pg of circular 3 kb plasmids containing ~500 bp of HBV or DHBV DNA were included as markers for the positions of dlDNA, and of rcDNA and cccDNA, respectively; a marker for ssDNA was obtained by heating the dlDNA marker to 95°C for 5 min.

**Isolation of cell nuclei by sucrose gradient sedimentation**

Purification of cell nuclei was done as described [5]. In brief, cells were harvested as described above and resuspended in 4.0 ml of low sucrose buffer (0.32 M sucrose, 3 mM CaCl_2_, 2 mM Mg^2+^ acetate, 1 mM DTT, 10 mM Tris-HCl [pH 8.0] and 0.5% Nonidet P-40). Subsequently, the cell lysate was mixed with 4.0 ml of high sucrose buffer (2.0 M sucrose, 5 mM Mg^2+^ acetate, 1 mM DTT, 10 mM Tris-HCl [pH 8.0]) and finally loaded on top of a cushion made of 4.4 ml high sucrose buffer. All steps were performed at 4°C. Centrifugation was done at 15,500 rpm at 4°C in a Beckman TST41 rotor for 45 minutes. The supernatant was subsequently removed by aspiration and the cell nuclei at the bottom of the tube were resuspended in 0.4 ml of lysis buffer. One half of the suspension was mixed with 30 units of micrococcal nuclease and incubated in the presence of 2 mM CaCl_2_ for 6 hours at 37°C before DNA extraction. The other half was processed for nucleic acid preparation without preceding micrococcal nuclease treatment and the purified DNA was digested with restriction enzyme Dpn I and Plasmid safe DNase prior to Southern blot analysis. An aliquot of the transfected cells was directly dissolved in lysis buffer without removal of cell nuclei and treated with micrococcal nuclease before DNA extraction by silica column adsorption; the procedure includes, as recommended by the manufacturer, a protease digestion step. Alternatively, DNA was prepared by phenol extraction. To this end, nuclear suspensions or cytoplasmic lysates were mixed with an equal volume of a 50 mM Tris-HCl [pH 8.0], 10 mM EDTA, 140 mM NaCl and 0.5% SDS containing buffer. The samples were incubated for 1 to 3 h in the presence of 1 mg/ml proteinase K at 56°C, treated with RNase A and passed through QIAshredder columns. Subsequently, DNA was extracted three times with Tris-buffered phenol/chloroform/isoamylalcohol (25/24/1/) and once with chloroform. After precipitation with ethanol the DNA was incubated with restriction enzyme Dpn I before Southern blot analysis. An aliquot of the samples was processed in the same way but without PK treatment. The white interphase containing non-digested proteins was carefully avoided during transfer of the aqueous phase.

**Distinction between nuclear rcDNA and nicked cccDNA**

Nicking of cccDNA is expected to occur at random locations in either strand; rcDNA, by contrast, is naturally discontinuous at defined positions (Figure 3A). The discontinuity in the plus-strand is located immediately upstream of direct repeat 2 (DR2; nt positions 2872-2882), leaving that region single-stranded to various extents, and therefore partially resistant to restriction enzyme digestion. Viral DNAs from isolated nuclei and from cytoplasmic extracts, treated with Dpn I to fragment the plasmid DNA, were restricted with Nco I (CCATGG; nt positions 2654-2659), Fsp I (TGCGCA; nt positions 3082-3087), and Apa LI (GTGCAC; nt positions 2861-2866; only 5 nt upstream of DR2), and analyzed by Southern blotting (Figure 3B). Cleavage by Fsp I and Apa LI is expected to split the HBV-internal 584 bp Dpn I fragment (nt positions 2683 to +85) into two subfragments of 400+184 bp (Fsp I), and 405+179 bp (Apa LI), respectively. In a separate experiment (Figure S5B), nuclear DNA was prepared either with, or without PK treatment and digested with Dpn I; only the sample not treated with PK was further digested with PsD. After removal of Dpn I and PsD, 50 pg of a DHBV plasmid was admixed to both samples before final restriction with Nco I, Fsp I or Apa LI. The blot was then successively probed with an HBV specific and a DHBV specific ^32^P labeled DNA probe.

**Cross-contamination analysis of gradient purified nuclei**

Cell nuclei were purified by sucrose gradient sedimentation as described above and dissolved in Laemmli loading buffer. An equivalent amount of whole cells was processed in the same way without gradient centrifugation to obtain a total cell lysate. Samples were passed through QIAshredder columns to reduce viscosity and loaded on 10% or 15% polyacrylamid gels. Proteins were transferred to Immobilon-P membranes (Millipore) and visualized with anti-PABP polyclonal antibody [6], or anti-histone H3 polyclonal antibody (Bethyl Laboratories) using the ECL Plus Western Blotting Detection System (GE Healthcare).

**Immunoprecipitation of viral nucleocapsids**

Nucleocapsids in cytoplasmic lysates were immunoprecipitated essentially as previously described [7], except that the lysates were adjusted to 0.75x RIPA [1x RIPA is 20 mM Tris (pH 7.2), 1 % sodium deoxycholate, 1 % Triton X-100, 0.1 % sodium dodecyl sulfate, 150 mM NaCl] to ensure comparable conditions with the nuclear lysates (see below). For HBV mab 312 [8], and for DHBV a polyclonal rabbit antiserum, 12/99, raised against recombinant DHBV capsids [7] were immobilized on protein G and protein A sepharose, respectively. Mock precipitations were performed using the DHBV specific antibodies for HBV transfected cells, and *vice versa*. For immunoprecipitation of nuclear capsids, the gradient-purified nuclear pellets, usually obtained from four 10 cm dishes of transfected cells, were treated with 0.75x RIPA buffer, which does not affect capsid integrity [9], and once sonicated (level 8, 50% intensity, 45 s) in a Branson sonifier 450 equipped with a cup resonator to achieve efficient release of the nuclear capsids; purely osmotic release [10] proved ineffective. The immunopellets were subsequently treated, or not, with MN and/or PK, and viral DNAs were isolated and analyzed as described above.

**Endogenous Polymerase Assay**

0.5 ml of HBV positive human serum (about 10^9^ viral genome equivalents [vge] per ml) or 1 ml of DHBV positive duck serum (about 10^10^ vge per ml) were loaded on top of a gradient made from 1.5 ml of 10% (w/v) Nycodenz and 0.4 ml of 50% Nycodenz (Nycomed Pharma) dissolved in lysis buffer containing 0.5% (v/v) NP-40 to remove the viral envelope. Centrifugation was done at 55,000 rpm for 45 minutes at 20°C using a Beckman TLS55 rotor. The fractions at the 10% to 50% boundary containing the viral capsids were collected, adjusted to 10 mM NH_4_Cl, 8 mM MgCl_2_, 0.06% β-mercaptoethanol and incubated for 16 h at 37°C in the presence of 1 mM each of dATP, dGTP, dCTP and dTTP. Aliquots were subsequently treated with 30 units micrococcal nuclease in the presence of 2 mM CaCl_2_ for 5 h at 37°C. Viral DNA was extracted with phenol after 1 h at 56°C in the presence or absence of PK, as described above.

**Quantitation and statistical evaluation of different viral DNA forms**

Signal intensities of individual bands on Southern blots were determined by phosphorimaging using a BAS1500 instrument and MacBas software (Fuji), or a Storm 865 imager and ImageQuant software (GE Healthcare). For background correction, values obtained for an equally sized area from the same lane were subtracted from the signal of interest. Values from each experiment were determined in duplicate. At least two, and often three or more independent experiments were evaluated in this way, as indicated in the text. To increase accuracy, in several experiments dilution series of a sample were used for relative comparisons. Proportionality of the amounts loaded and the signal intensities was confirmed by linear regression analysis (Graphpad Prism 5 for Mac). Mean values and standard deviations (SD) were calculated using Microsoft Excel software; where appropriate, values in the text are given as mean ± SD. Absolute amounts of a given species per sample were derived by normalization to known amounts (usually 10 pg per lane, equivalent to ~3x10^6^ viral genomes) of the dlDNA marker (see above). Copy numbers per cell were calculated from the number of cells per dish, or for samples containing chromosomal DNA by measuring the absorbance at 260 nm and assuming a DNA content of 6.5 pg per diploid cell; copy numbers per transfected cell were derived by correcting for transfection efficiency which was routinely around 20%, as determined by transfections with a GFP encoding plasmid. Statistical significance of differences between different groups were evaluated using Graphpad Prism 5 for Mac.

**REFERENCES**

1. Halverscheid L, Mannes NK, Weth R, Kleinschmidt M, Schultz U, et al. (2008) Transgenic mice replicating hepatitis B virus but lacking expression of the major HBsAg. J Med Virol 80: 583-590.

2. Nassal M (1992) The arginine-rich domain of the hepatitis B virus core protein is required for pregenome encapsidation and productive viral positive-strand DNA synthesis but not for virus assembly. J Virol 66: 4107-4116.

3. Lenhoff RJ, Summers J (1994) Coordinate regulation of replication and virus assembly by the large envelope protein of an avian hepadnavirus. J Virol 68: 4565-4571.

4. Schlicht HJ, Radziwill G, Schaller H (1989) Synthesis and encapsidation of duck hepatitis B virus reverse transcriptase do not require formation of core-polymerase fusion proteins. Cell 56: 85-92.

5. Marzluff WF, Jr. (1978) Transcription of RNA in isolated nuclei. Methods Cell Biol 19: 317-332.

6. Hundsdoerfer P, Thoma C, Hentze MW (2005) Eukaryotic translation initiation factor 4GI and p97 promote cellular internal ribosome entry sequence-driven translation. Proc Natl Acad Sci U S A 102: 13421-13426.

7. Vorreiter J, Leifer I, Rösler C, Jackevica L, Pumpens P, et al. (2007) Monoclonal antibodies providing topological information on the duck hepatitis B virus core protein and avihepadnaviral nucleocapsid structure. J Virol 81: 13230-13234.

8. Sällberg M, Ruden U, Magnius LO, Harthus HP, Noah M, et al. (1991) Characterisation of a linear binding site for a monoclonal antibody to hepatitis B core antigen. J Med Virol 33: 248-252.

9. Cao F, Tavis JE (2004) Detection and characterization of cytoplasmic hepatitis B virus reverse transcriptase. J Gen Virol 85: 3353-3360.

10. Muckenfuss H, Kaiser JK, Krebil E, Battenberg M, Schwer C, et al. (2007) Sp1 and Sp3 regulate basal transcription of the human APOBEC3G gene. Nucleic Acids Res 35: 3784-3796.
